# Genetic and biochemical analyses reveal direct interactions between LitR and genes important for *Vibrio fischeri* physiology, including biofilm production

**DOI:** 10.1128/jb.00042-25

**Published:** 2025-08-01

**Authors:** Brittany L. Fung, Chase Mullins, Douglas B. Rusch, Elizabeth G. Musto, Julia C. van Kessel, Karen L. Visick

**Affiliations:** 1 Department of Microbiology and Immunology, Loyola University Chicago 548051, Maywood, Illinois, USA; 2 Department of Biology, Indiana University 1772, Bloomington, Indiana, USA; 3 Center for Genomics and Bioinformatics, Indiana University 1772, Bloomington, Indiana, USA; Geisel School of Medicine at Dartmouth, Hanover, New Hampshire, USA

**Keywords:** *Vibrio fischeri*, quorum sensing, biofilms, cellulose, adhesins

## Abstract

Bacteria can link gene expression to population density to promote group behaviors using quorum sensing. Quorum sensing controls a multitude of bacterial processes, such as virulence, motility, and biofilm formation. In *Vibrio fischeri*, the quorum-sensing-dependent transcription factor LitR inhibits biofilm formation. A previous study showed that LitR inhibits transcription (~1.4-fold) of the *bcs* locus, which comprises the genes responsible for producing the cellulose polysaccharide. However, beyond that, the mechanism of LitR-mediated inhibition of biofilm formation was unknown. Here, we find that LitR transcriptionally activates *pdeV*, which encodes a c-di-GMP phosphodiesterase that indirectly promotes cleavage of the large adhesive protein LapV from the surface of *V. fischeri*, leading to biofilm dispersal. LitR also induces transcription of the gene for sensor kinase VF_A1016, which we determined to be important for biofilm inhibition. Like the loss of LitR, the loss of VF_A1016 increased *bcs* transcription (~1.6-fold). Through chromatin immunoprecipitation sequencing (ChIP-seq), we found that LitR directly binds to the *VF_A1016* and *pdeV* regulatory region. In total, we identified 147 LitR-binding sites in the genome and confirmed transcriptional control over a subset of these putative regulatory targets. Specifically, we determined that LitR induces transcription of the genes encoding the diguanylate cyclase VF_1200 and the glyoxylate shunt protein AceB and inhibits expression of the putative transcription factor TfoY. These data expand our understanding of LitR-mediated regulation of genes involved in biofilm formation and the physiology of *V. fischeri*.

**IMPORTANCE:**

Bacteria can coordinate their behaviors on a population level using quorum sensing, a process that results in altered gene regulation. In *Vibrio fischeri*, the quorum-sensing-regulated transcription factor LitR inhibits the formation of biofilms, communities of attached and protected bacteria, by diminishing the production of cellulose. Here, we determined that LitR controls additional known or putative biofilm factors. We also identified other possible targets of LitR regulation by high-throughput chromatin immunoprecipitation sequencing. This work furthers our understanding of the established connection between quorum sensing and biofilm formation in *V. fischeri* strain ES114. These findings also have the potential to translate to known pathways in other *Vibrios* where quorum sensing and biofilm production are linked.

## INTRODUCTION

Bacteria can alter their gene expression at a population level, leading to multicellular-like behavior (1). This process, called quorum sensing (2), occurs when bacterial cells release and then detect small molecules (or peptides) called autoinducers. Under high cell density conditions, autoinducers activate signaling cascades that control the regulation of downstream genes, leading to behaviors such as competition, competence, biofilm formation, and bioluminescence (3).

**Fig 1:**
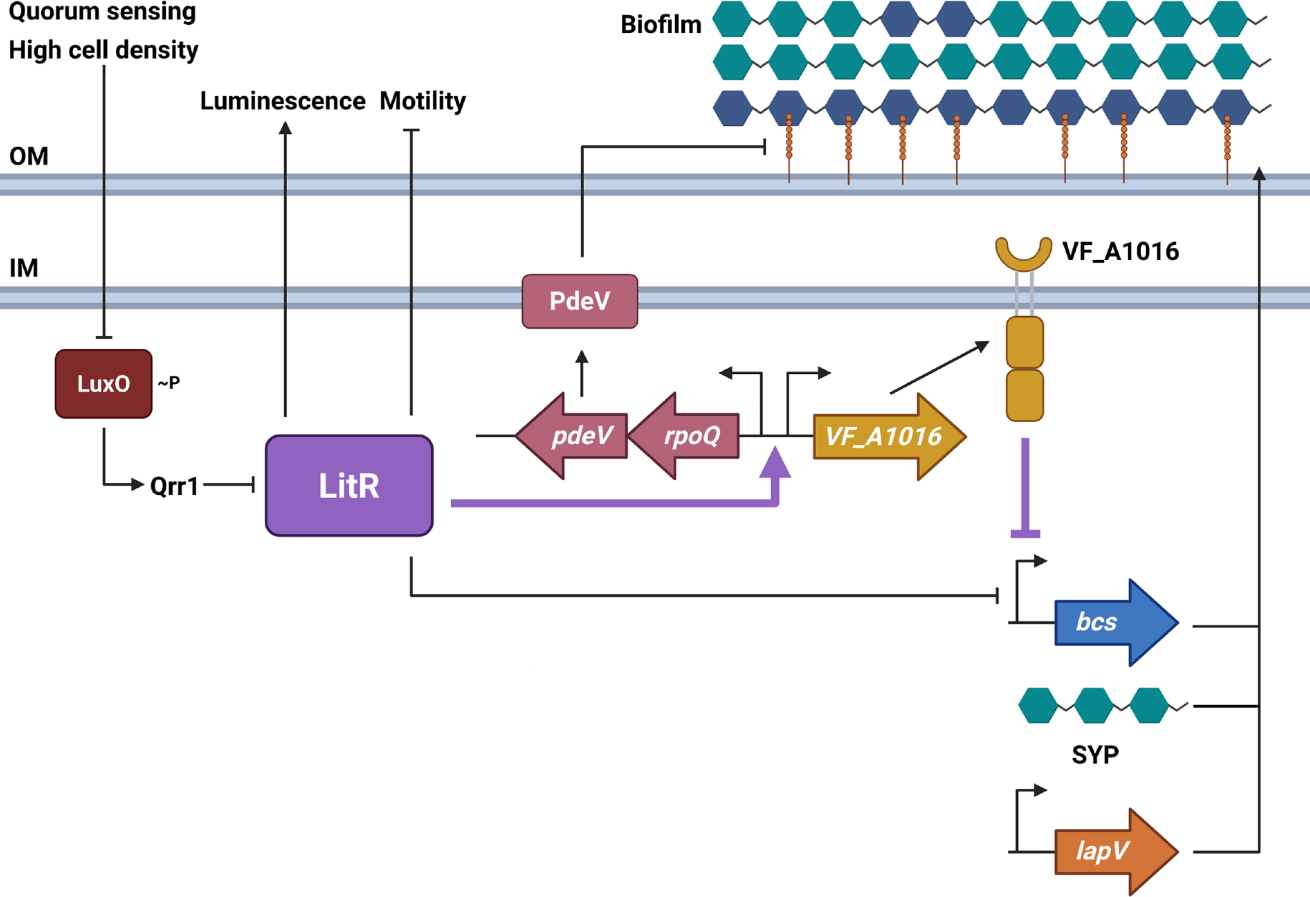
The known LitR pathway and relevant genes. At high cell densities, the quorum-sensing regulator LuxO is dephosphorylated, preventing Qrr1 expression and thus allowing for LitR production. LitR induces luminescence and inhibits motility. *V. fischeri* biofilms are composed of SYP (teal hexagons), cellulose polysaccharide (blue hexagons), and the LapV adhesin (orange line). Cellulose and LapV are produced by the *bcs* locus and *lapV* gene, respectively. LitR modulates biofilm formation by inhibiting *bcs* transcription. LitR also increases transcriptional activity of *pdeV* and *VF_A1016* by directly binding to their regulatory region. PdeV is a phosphodiesterase whose activity indirectly results in cleavage of LapV from the surface of *V. fischeri*, leading to biofilm dispersal. The sensor kinase VF_A1016 also inhibits *bcs* transcription to control biofilm formation. Bolded/purple lines indicate connections uncovered in this work. OM, outer membrane; IM, inner membrane. Created in BioRender. Fung, B. (2025) https://BioRender.com/e94v572.

*Vibrio fischeri*, a marine microorganism, uses quorum sensing to control bioluminescence, competence, motility, the type VI secretion system, and acetate metabolism (4–12). At low cell densities, LuxO, a quorum-sensing-controlled response regulator, is phosphorylated and active, leading to the expression of the sRNA Qrr1 (5, 13, 14) (Fig. 1). Qrr1 inhibits *litR* translation (5); thus, LitR levels are low, and regulation of downstream genes is minimal. LitR is a conserved TetR-type transcriptional regulator found in other *Vibrios* such as HapR in *Vibrio cholerae* and LuxR in *Vibrio harveyi* (4, 15, 16). When the bacteria reach a high cell density, autoinducers bind to their receptors at a higher frequency, leading to the dephosphorylation of LuxO, thus preventing the activation of the sRNA Qrr1 (Fig. 1). This leads to increased *litR* mRNA translation (5) and an increase in LitR-dependent phenotypes.

A recent study expanded the known function of LitR to include the ability to inhibit the formation of biofilms, attached and protected microbial communities (17, 18). *V. fischeri* biofilms can contain both symbiosis polysaccharide (SYP) and cellulose polysaccharide, with SYP contributing to biofilm cohesion and cellulose allowing for surface attachment (19–22). The relative importance of the two polysaccharides varies depending on the growth conditions. Generally, biofilms depend on the production of both SYP and cellulose polysaccharides under static growth conditions, but are more heavily dependent on cellulose under shaking conditions (18). Loss of LitR increases biofilm formation under both conditions (18). This phenotype could be due, at least in part, to LitR-mediated inhibition of *bcs* (cellulose) transcription observed under shaking conditions.

In addition to SYP and cellulose polysaccharide, another component of the *V. fischeri* biofilm is LapV, a surface adhesin that likely mediates the connection between cells and polysaccharide components and/or surfaces (23). The presence of LapV on the surface of *V. fischeri* is indirectly modulated by the levels of cyclic diguanylate (c-di-GMP) (23–26). Currently, a single c-di-GMP phosphodiesterase (PDE), PdeV, has been implicated in diminishing the c-di-GMP levels needed for maintaining surface-associated LapV (23). Thus, it was notable that a previous microarray identified *pdeV*, among other genes, as negatively regulated by LuxO (7), the upstream inhibitor of LitR (Fig. 1).

Here, we further probed the role of LitR in controlling biofilm formation. We determined that LitR induces transcription both of *pdeV,* likely leading to cleavage of LapV, and of *VF_A1016*, which encodes a sensor kinase that, in turn, inhibits the transcription of cellulose genes. We also used chromatin immunoprecipitation (ChIP-seq) to globally identify LitR-regulated genes, which confirmed that LitR directly controls *pdeV* and *VF_A1016* transcription. Furthermore, 147 binding sites and 181 putative LitR-controlled genes were identified. For the diguanylate cyclase VF_1200, the glyoxylate shunt enzyme AceB, and the putative transcription factor TfoY (6, 11, 27–30), we confirmed that LitR exerts control at the level of transcription. Together, our data provide potential explanations for a number of LitR-controlled phenotypes, including biofilm formation, furthering our understanding of the role of this important regulator in the physiology of *V. fischeri*.

## RESULTS

### The ∆*litR* mutant biofilm is dependent on the presence of LapV

**Fig 2:**
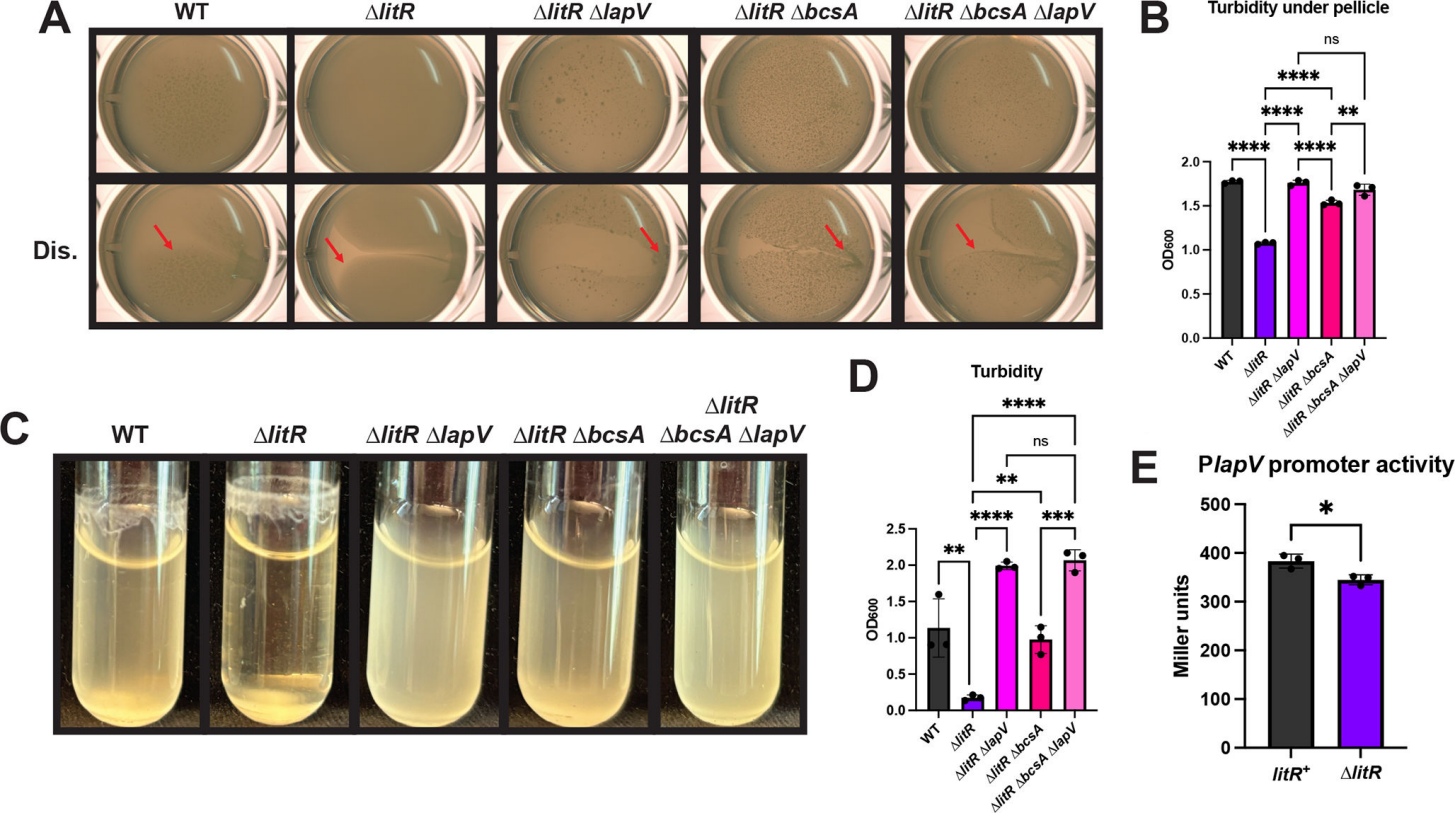
The ∆*litR* mutant is dependent on LapV for biofilm formation. (**A**) WT (ES114), the ∆*litR* mutant (KV10494), the ∆*litR* ∆*lapV* mutant (BF76), the ∆*litR* ∆*bcsA* mutant (BF11), and the ∆*litR* ∆*bcsA* ∆*lapV* mutant (BF669) were assessed after 72 h of static growth at 24°C in LBS + 10 mM CaCl_2_. Pellicles were imaged using the Zeiss Stemi 2000-c microscope at 6.5× magnification with and without disruption (Dis.) using a toothpick to assess stickiness of the pellicle. Red arrows are used to highlight an area of each disrupted pellicle where stickiness and/or cohesiveness is observed. (**B**) Turbidity of the liquid underneath the pellicle was measured by OD_600_ and plotted. (**C**) WT (ES114), the ∆*litR* mutant (KV10494), the ∆*litR* ∆*lapV* mutant (BF76), the ∆*litR* ∆*bcsA* mutant (BF11), and the ∆*litR* ∆*bcsA* ∆*lapV* mutant (BF669) were imaged after 24 h of shaking growth at 24°C in tTBS + 10 mM CaCl_2_. (**D**) The turbidity of the liquid was measured by OD_600_ and plotted. (**E**) The *litR*^+^ strain (BF598) and the ∆*litR* mutant (BF599), both carrying a ∆*sypQ* mutation and the P*lapV-lacZ* reporter, were grown with shaking in LBS + 10 mM CaCl_2_ at 24°C for 22 h. Cell extracts were then assessed for β-galactosidase activity as a measure of P*lapV* activity, and the final Miller units were calculated. Statistics for panels B and D were performed using a one-way analysis of variance (ANOVA) corrected for multiple comparisons with Tukey’s test; ns: not significant, **P-value < 0.0063, ***P-value: 0.0006, ****P-value < 0.0001. Statistics for panel E were performed using an unpaired t-test; *P-value: 0.0193.

We previously determined that LitR caused a ~1.4× decrease in transcription of the cellulose genes, an amount that seemed inadequate to fully explain the negative impact of LitR on biofilm formation (18). Thus, we explored a role for LitR in controlling other biofilm factors, such as the LapV adhesin. As previously observed, the ∆*litR* mutant produced a more robust biofilm compared to WT under both static and shaking liquid conditions (18) (Fig. 2A-D). Under static conditions, the pellicle was visually more cohesive and, correspondingly, the turbidity underneath the pellicle was lower in comparison to WT (Fig. 2A and B). In shaking liquid conditions, the ∆*litR* mutant showed a decrease in turbidity and formed a larger clump at the bottom of the culture (Fig. 2C and D). Loss of LapV diminished both these phenotypes. In static growth, the ∆*litR* ∆*lapV* mutant pellicle was thinner, with very slight stickiness, and the liquid underneath was more turbid than that of its Δ*litR* parent (Fig. 2A and B). Moreover, in shaking conditions, the culture was completely turbid and no ring was apparent, indicating that the biofilm produced by the ∆*litR* mutant is fully dependent on *lapV* under these conditions (Fig. 2C and D).

Because the biofilms formed under both static and shaking conditions also depend on cellulose polysaccharide (18), we next investigated the relative contributions of cellulose and LapV for these biofilm phenotypes. In static growth, the ∆*litR* ∆*bcsA* mutant was slightly less turbid compared to the ∆*litR* ∆*lapV* double mutant and the triple mutant (Fig. 2A and B), which indicates that LapV makes a greater contribution to biofilm formation in this condition. During growth with shaking, loss of LapV resulted in the greatest turbidity (least biofilm); the ∆*litR* ∆*bcsA* ∆*lapV* mutant phenocopied the ∆*litR* ∆*lapV* mutant (Fig. 2C and D). These data indicate that both factors make key contributions, but LapV makes a greater contribution to these phenotypes than cellulose. We hypothesize that LapV may facilitate the function of cellulose and potentially a second factor, likely SYP polysaccharide, to promote biofilm formation under these conditions.

Because LitR is a transcription factor, we asked whether LitR inhibits *lapV* transcription to negatively regulate LapV production. We tested this possibility using a β-galactosidase assay with a strain that carried the *lapV* promoter fused to a promoterless *lacZ* gene. The loss of LitR exerted a minimal but significant negative effect (Fig. 2E); introduction of the *litR*-expressing multi-copy plasmid pPMF5 into the ∆*litR* mutant correspondingly caused an increase in transcription (Fig. S1A). These data indicate that LitR increases, rather than decreases, P*lapV* promoter activity. Because this result was inconsistent with our phenotypic assays, we subsequently pursued the possibility that LitR may inhibit LapV activity in an alternate manner under these conditions.

### LitR controls *pdeV* transcription to inhibit biofilm formation

**Fig 3:**
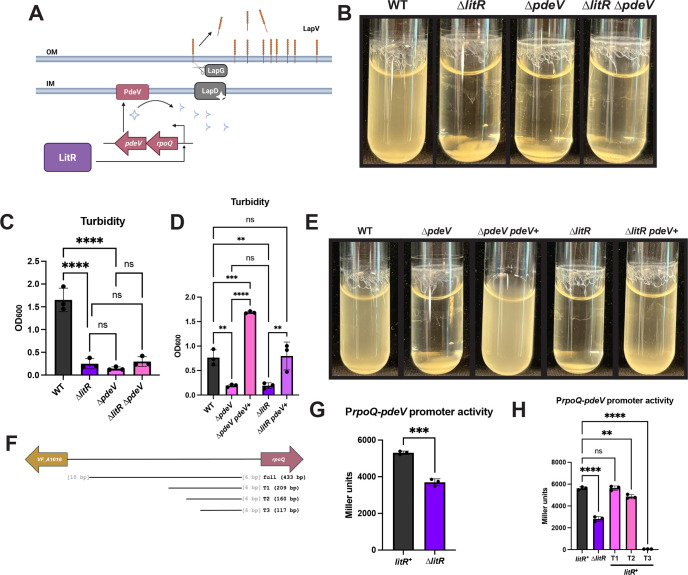
LitR controls *pdeV* transcription to impact biofilm formation. (**A**) A model of control over LapV cleavage in *V. fischeri*, based on work in *P. fluorescens* (31–35) and *V. fischeri* (23). When PdeV is active, it degrades c-di-GMP (light blue diamonds). When there are low levels of c-di-GMP, LapD is unable to bind to LapG with high affinity, allowing LapG to cleave LapV (orange line) from the surface of the cell. LitR induces *rpoQ* and *pdeV* transcription. OM, outer membrane; IM, inner membrane. The following strains were imaged after 24 h of shaking growth in tTBS + 10 mM calcium at 24°C: (**B**) WT (ES114), the ∆*litR* mutant (KV10494), the ∆*pdeV* mutant (KV8969), the ∆*litR* ∆*pdeV* mutant (BF607), (**E**) WT (ES114), the ∆*pdeV* mutant (KV8969), the ∆*pdeV* mutant with P*nrdR-RBS-pdeV-flag* (*pdeV*^+^) (KV9918), the ∆*litR* mutant (KV10494), and the ∆*litR pdeV*^+^ strain (BF91). (**C** and **D**) Turbidity of the liquid was measured by OD_600_ and plotted. (**F**) A cartoon showing the full-length *VF_A1016-rpoQ* regulatory region used in the P*rpoQ-pdeV* β-galactosidase assays and the subsequent truncations (T). The letters in gray represent the number of bases between the region of interest and the start of *rpoQ* or *VF_A1016*. (**G**) The *litR*^+^ strain (BF297) and the ∆*litR* mutant (BF298), both carrying a ∆*sypQ* mutation and the P*rpoQ-pdeV-lacZ* reporter, (**H**) along with the *litR*^+^ strain with a ∆*sypQ* mutation and the P*rpoQ-pdeV-lacZ* reporter truncated to the bp listed in panel F, T1 (KV10670), T2 (KV10803), and T3 (KV10867), were tested after 22 h of shaking growth at 24°C in LBS + 10 mM CaCl_2_. Cell extracts were assessed for β-galactosidase activity as a measure of P*rpoQ-pdeV* activity, and the final Miller units were calculated. Statistics for panels C, D, and H were performed using a one-way ANOVA corrected for multiple comparisons with Tukey’s test; ns: not significant, **P-value < 0.0059, ***P-value: 0.0001, ****P-value < 0.0001. Statistics for panel G were performed using an unpaired t-test; ***P-value: 0.0002. Created in BioRender. Fung, B. (2025) https://BioRender.com/e94v572.

Previous work showed that the surface localization of LapV is controlled through a c-di-GMP-dependent mechanism (23) (Fig. 3A). More specifically, the PDE PdeV removes the c-di-GMP needed for maintenance of LapV on the surface (23, 31–35). We thus hypothesized that because LitR does not inhibit transcription of LapV, it could influence LapV function by controlling PdeV and thus c-di-GMP levels. To begin to address this question, we asked whether these proteins functioned in the same pathway by evaluating the biofilm phenotypes of mutants that were deleted for *pdeV* alone or in combination with a deletion of *litR*. As previously observed (23), the Δ*pdeV* mutant exhibited increased biofilm formation under shaking conditions that could be indirectly quantified by a decrease in turbidity that was not due to a growth defect (Fig. 3B and C; Fig. S2). We did not observe any increase in biofilm formation by the double ∆*litR* Δ*pdeV* mutant; it phenocopied the single Δ*pdeV* and Δ*litR* mutants for biofilm formation (Fig. 3B and C). These data support, but do not confirm, the possibility that LitR and PdeV could act in the same pathway to inhibit biofilm formation under these conditions.

To evaluate this possibility further, we examined biofilm formation by a derivative of the Δ*litR* mutant that carried a second copy of *pdeV*, one that is controlled by the non-native promoter, P*nrdR*, which has recently been used for expression of other genes (36). When the strains were grown with shaking, the additional copy of *pdeV* was able to suppress the ∆*litR* mutant phenotype to approximately WT levels of turbidity (Fig. 3D and E). These results support the conclusion that LitR and PdeV are in the same pathway for biofilm inhibition under shaking conditions, and PdeV is epistatic to LitR.

Lastly, we hypothesized that LitR positively regulates *pdeV* transcription. *pdeV* is positioned downstream of *rpoQ* in an apparent operon (Fig. 1 and 3A). We first used reverse transcriptase polymerase chain reaction (RT-PCR) to determine whether these genes are co-transcribed. After reverse-transcribing the RNA to DNA, we obtained bands corresponding to *rpoQ*, *pdeV*, and the intergenic region between the two genes (Fig. S3). These data indicate that *rpoQ* and *pdeV* are transcribed as a single transcript.

Therefore, we fused the regulatory region upstream of *rpoQ* to a promoterless *lacZ* gene and evaluated the resulting strain using β-galactosidase assays. We found that deletion of *litR* decreased promoter activity from P*rpoQ-pdeV* (Fig. 3G). We then assessed the ∆*litR* mutant carrying the *litR*-expressing plasmid for β-galactosidase activity, which increased, relative to the vector control, in P*rpoQ-pdeV lacZ* reporter activity (Fig. S1B), confirming that LitR exerts a positive impact on transcription from this promoter. These data correspond to previous microarray analyses where *rpoQ* and *pdeV* transcription were increased in a strain deleted for a negative regulator of *litR*, LuxO (7) (Fig. 1), and RT-quantitative PCR (RT-qPCR) results in which the loss of LitR resulted in decreased levels of *rpoQ* transcripts (10). Furthermore, we found that introduction into *Escherichia coli* of both pPMF5 and a plasmid that contains the P*rpoQ-pdeV lacZ* reporter similarly caused an increase in β-galactosidase activity relative to the vector-containing control strain (Fig. S1C). The finding that LitR impacts transcription in this heterologous system supports the conclusion that LitR directly impacts transcription.

To indirectly examine whether LitR binds to the *rpoQ* regulatory region, we truncated the region and assessed transcriptional activity to determine whether any truncation would result in a decrease in promoter activity to the levels of the ∆*litR* mutant. Truncation of the *rpoQ* regulatory region from the full-length 433 bp fragment to 160 bp (T2) led to a significant loss in P*rpoQ-pdeV* activity, though not to the extent of the ∆*litR* mutant. Complete loss of promoter activity occurred when only 117 bp was present (T3), suggesting that we truncated within or beyond the promoter sequence or the binding site of a required activator (Fig. 3F and H). Based on these data, LitR, or a different activator, may bind sequences positioned between the first and second truncations within the *rpoQ* regulatory region to activate *rpoQ* and *pdeV* transcription. Taken together, LitR increases *rpoQ* and thus, *pdeV* transcription, presumably resulting in increased levels of PdeV that, in turn, can inhibit biofilm formation (Fig. 3A).

**Fig 4:**
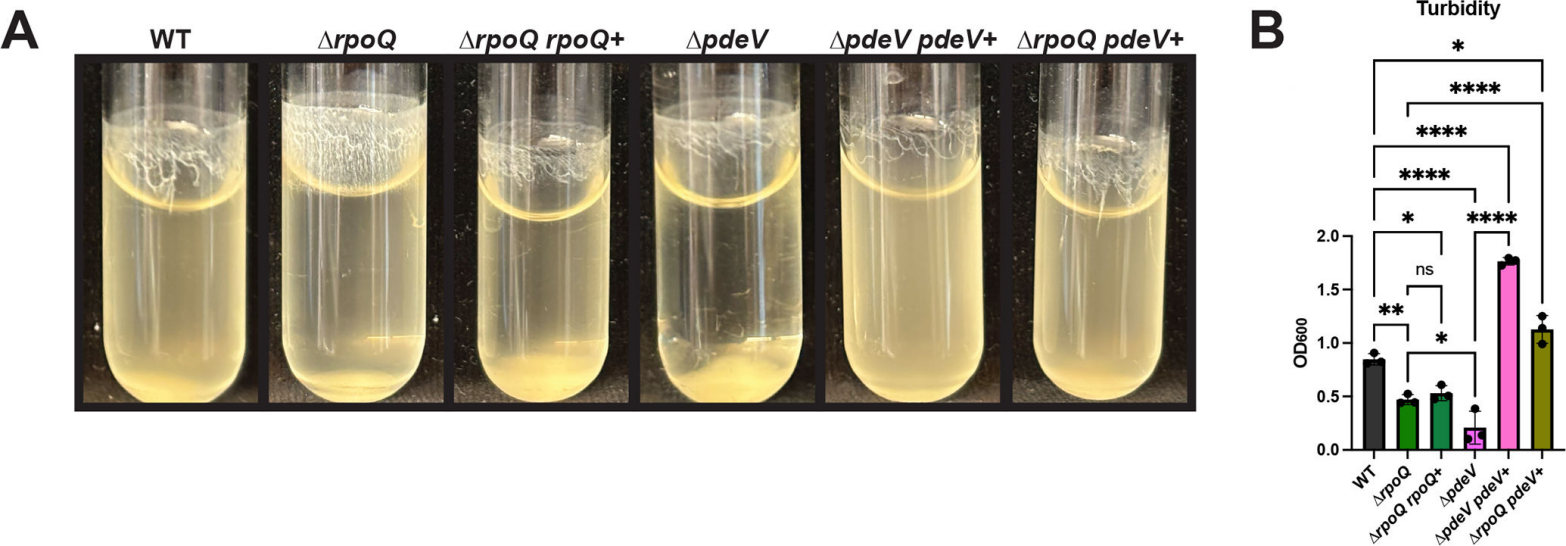
The ∆*rpoQ* mutation is polar on *pdeV*. (**A**) WT (ES114), the ∆*rpoQ* mutant (BF49), the ∆*rpoQ* mutant with P*rpoQ-rpoQ* (*rpoQ*^+^) (KV10931), the ∆*pdeV* mutant (KV8969), the ∆*pdeV* ∆*pdeV*^+^ strain (KV9918), and the ∆*rpoQ pdeV*^+^ strain (KV10932) were imaged after 24 h of shaking growth in tTBS + 10 mM CaCl_2_ at 24°C. (**B**) The turbidity of the liquid was measured by OD_600_ and plotted. Statistics were performed using a one-way ANOVA corrected for multiple comparisons with Tukey’s test; ns: not significant, *P-value < 0.0432, **P-value: 0.0034, ****P-value < 0.0001.

Because LitR induces transcription of the *rpoQ-pdeV* operon (Fig. 3G; Fig. S1B and C) (10), we wondered whether RpoQ also contributed to biofilm formation; previous work had only demonstrated motility, chitinase, and luminescence phenotypes for RpoQ overexpression (10). The ∆*rpoQ* mutant had increased shaking biofilm formation compared to WT (Fig. 4). However, this phenotype could not be complemented by inserting *rpoQ* at a non-native site within the chromosome, indicating that the *rpoQ* deletion may be polar on *pdeV*. Indeed, the biofilm phenotypes of the Δ*rpoQ* mutant could be suppressed by overexpression of *pdeV* (Fig. 4), suggesting that loss of PdeV is responsible for the ∆*rpoQ* mutant biofilm phenotype. Overall, we conclude that *rpoQ* and *pdeV* form an operon, and that PdeV inhibits *V. fischeri* biofilm formation.

### LitR controls *VF_A1016* transcription to inhibit biofilm formation

Divergently transcribed from the *rpoQ-pdeV* operon is another gene of interest, *VF_A1016* (Fig. 1). *VF_A1016* was shown through RT-qPCR analysis to be inhibited by LuxO at an OD_600_ of 0.5 (10) and encodes a sensor kinase (20, 37). Together, these findings regarding *VF_A1016—*the chromosomal location and regulation by LuxO—suggested that VF_A1016 could be regulated by LitR, potentially to contribute to biofilm inhibition.

**Fig 5:**
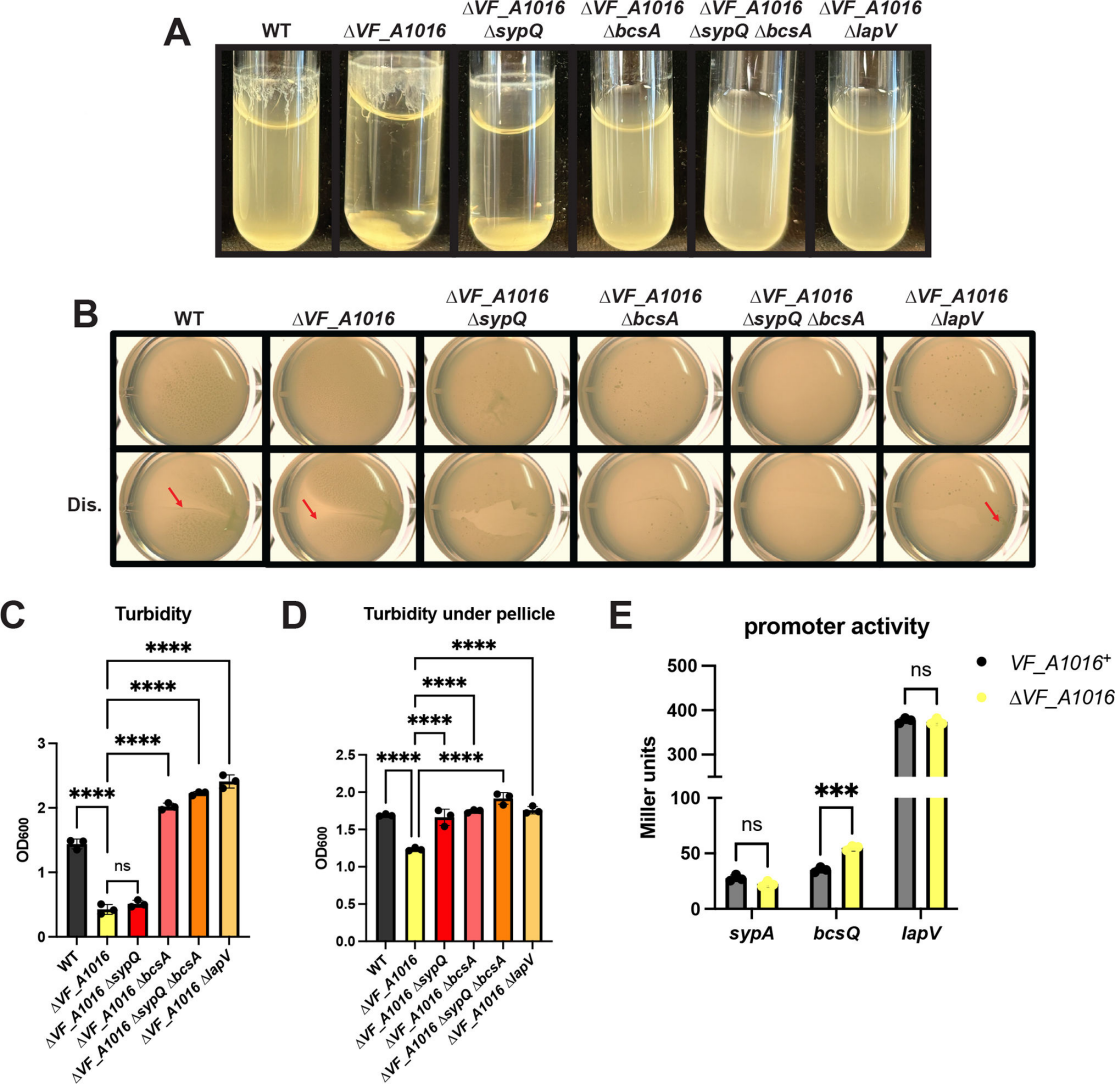
VF_A1016 inhibits biofilm formation. WT (ES114), the ∆*VF_A1016* mutant (BF48), the ∆*VF_A1016* ∆*sypQ* mutant (BF84), the ∆*VF_A1016* ∆*bcsA* mutant (BF85), the ∆*VF_A1016* ∆*sypQ* ∆*bcsA* mutant (BF109), and the ∆*VF_A1016* ∆*lapV* mutant (BF89) were incubated at 24°C and imaged after (**A**) 24 h of shaking growth in tTBS + 10 mM CaCl_2_ or (**B**) 72 h of static growth in LBS + 10 mM CaCl_2_. Pellicles were imaged with or without disruption (Dis.) using the Zeiss Stemi 2000-c microscope at 6.5× magnification. Disruption was done using a toothpick to assess the stickiness of the pellicle. Red arrows are used to highlight an area of each disrupted pellicle where stickiness and/or cohesiveness is observed. Turbidity of (**C**) the shaking biofilm liquid and (**D**) the liquid underneath the pellicle was measured by OD_600_ and plotted. (**E**) *VF_A1016*^+^ and the ∆*VF_A1016* mutant, both carrying a ∆*sypQ* mutation and the P*sypA-lacZ* reporter (*sypA*) (BF237 and BF244), the P*bcsQ-lacZ* reporter (*bcsQ*) (BF255 and BF259), or the P*lapV-lacZ* reporter (*lapV*) (BF598 and BF609) were grown with shaking for 22 h at 24°C in LBS + 10 mM CaCl_2_. Cell extracts were then assessed for β-galactosidase activity as a measure of reporter activity, and the final Miller units were calculated. Statistics for panels C and D were performed using a one-way ANOVA corrected for multiple comparisons using Tukey’s test; ns: not significant, ****P-value < 0.0001. Statistics for panel E were performed using a two-way ANOVA using Šídák’s multiple comparison test; ns: not significant, ***P-value: 0.0003.

To test this possibility, we first analyzed biofilm formation by a Δ*VF_A1016* mutant in shaking and static conditions. In both assays, the ∆*VF_A1016* mutant exhibited increased biofilm formation and decreased turbidity compared to WT (Fig. 5A-D), which was not due to a growth defect (Fig. S2), suggesting that VF_A1016 inhibits biofilm formation. Like the phenotypes of the Δ*litR* mutant (18) (Fig. 2A-D), the increased biofilm phenotypes of the Δ*VF_A1016* mutant required cellulose and LapV (Fig. 5A-D). In addition to cellulose and LapV, the static liquid phenotype depended on SYP (Fig. 5B and D).

To determine whether VF_A1016 controlled transcription of any of these genes, we evaluated β-galactosidase activity of representative promoter-*lacZ* fusions in the presence and absence of VF_A1016. Loss of VF_A1016 exerted an effect only on the transcription of *bcs* (~1.6-fold) (Fig. 5E). These results suggest that regulation of biofilm formation by VF_A1016 may occur, at least in part, by negative control over transcription of the cellulose locus.

**Fig 6:**
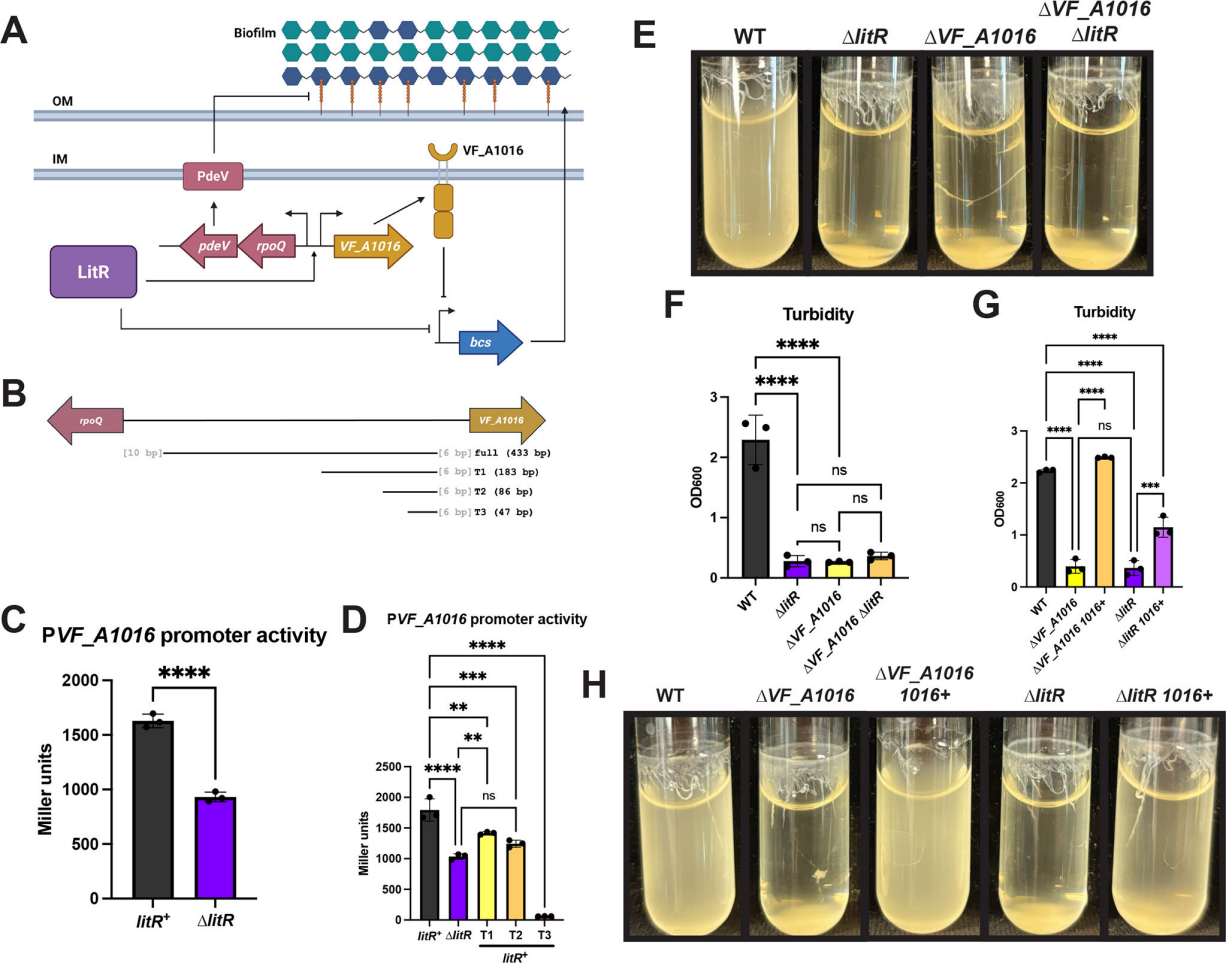
LitR controls *VF_A1016* transcription to impact biofilm formation. (**A**) A cartoon depicting LitR regulation of *V. fischeri* biofilms via PdeV and VF_A1016. LitR activates *pdeV* and *VF_A1016* transcription, leading to production of PdeV and VF_A1016, respectively. PdeV inhibits the presence of LapV (orange line) on the surface of the cell. LitR and VF_A1016 both inhibit *bcs* (cellulose) transcription, leading to reduced cellulose (blue hexagons) production. SYP (teal hexagons) is another contributor to *V. fischeri* biofilms. OM, outer membrane; IM, inner membrane. (**B**) A cartoon showing the full-length *VF_A1016-rpoQ* regulatory region used in the P*VF_A1016* β-galactosidase assays and the subsequent truncations (T). The letters in gray represent the number of bases between the region of interest and the start of *rpoQ* or *VF_A1016*. (**C**) The *litR*^+^ strain (BF299) and the ∆*litR* mutant (BF310), both carrying a ∆*sypQ* mutation and the P*VF_A1016-lacZ* reporter, or (**D**) along with the *litR*^+^ strain with a ∆*sypQ* mutation and the P*VF_A1016-lacZ* reporter truncated to the listed bp in panel B, T1 (KV10775), T2 (KV10804), and T3 (KV10865), were tested after 22 h of shaking growth at 24°C in LBS + 10 mM CaCl_2_. Cell extracts were assessed for β-galactosidase activity as a measure of P*VF_A1016* activity, and the final Miller units were calculated. (**E**) WT (ES114), the ∆*litR* mutant (KV10494), the ∆*VF_A1016* mutant (BF48), and the ∆*VF_A1016* ∆*litR* mutant (BF204) or (**H**) WT (ES114), the ∆*VF_A1016* mutant (BF48), the ∆*VF_A1016* mutant with P*nrdR-VF_A1016* (*1016*^+^) (BF203), the ∆*litR* mutant (KV10494), and the ∆*litR 1016*^+^ strain (BF212) were imaged after 24 h of shaking growth at 24°C in tTBS +10 mM CaCl_2_. (**F** and **G**) Turbidity of the liquid was measured by OD_600_ and plotted. Statistics for panel C were performed using an unpaired t-test; ****P-value < 0.0001. Statistics for panels D, F, and G were performed using a one-way ANOVA corrected for multiple comparisons with Tukey’s test; ns: not significant, **P-value < 0.0027, ***P-value: 0.0001, ****P-value < 0.0001. Created in BioRender. Fung, B. (2025) https://BioRender.com/e94v572.

Next, we more directly asked if LitR could promote transcription of VF_A1016, as suggested by the previous RT-qPCR analyses of a ∆*luxO* mutant (10), using a P*VF_A1016-lacZ* reporter construct encompassing the intergenic region between *rpoQ* and *VF_A1016*. We found that the ∆*litR* mutant exhibited decreased β-galactosidase activity from P*VF_A1016-lacZ* compared to its *litR*^+^ parent (Fig. 6C). This defect in β-galactosidase activity was complemented by the introduction of *litR*-expression plasmid pPMF5 (Fig. S1D). These results suggest that LitR activates *VF_A1016* transcription (Fig. 6A). Further supporting this conclusion and suggesting that this effect is mediated directly by LitR, the introduction of pPMF5 and a plasmid that contained the P*VF_A1016-lacZ* reporter into *E. coli* similarly caused increased β-galactosidase activity relative to the corresponding vector control strain (Fig. S1E).

We evaluated the regulatory region by making deletions. Truncating the *VF_A1016* regulatory region from the full-length 433 bp fragment to 183 bp (T1) and 86 bp (T2) resulted in a significant and stepwise decrease in *VF_A1016* promoter activity (Fig. 6B and D), suggesting that the binding site for LitR, or that for another activator, was lost. Lastly, truncating the region to 47 bp (T3) significantly impacted P*VF_A1016* activity, potentially indicating that this truncation directly affected the *VF_A1016* promoter.

We genetically assessed whether LitR is in the same pathway as VF_A1016 for biofilm inhibition by double mutant and suppression analyses. We constructed and evaluated the double ∆*VF_A1016* ∆*litR* mutant, which phenocopied its single mutants (Fig. 6E and F), supporting our hypothesis that LitR and VF_A1016 are in the same pathway. We also asked whether we could suppress the ∆*litR* mutant phenotype by making transcription of VF_A1016 independent of LitR. To do so, we introduced a second copy of *VF_A1016* at a non-native site under the control of P*nrdR*. There was a small suppression of the biofilms formed in shaking conditions, primarily visualized through the quantifiable increase in turbidity (Fig. 6G and H). Overall, these data align with a model in which LitR acts upon P*VF_A1016* to inhibit biofilm formation, potentially in a cellulose-dependent manner, under shaking growth conditions (Fig. 6A).

### Co-expression of both *VF_A1016* and *pdeV* is required to restore WT-like biofilm formation to the ∆*litR* mutant under static conditions

Above, we primarily assessed the contributions of PdeV and VF_A1016 to LitR-dependent biofilms under shaking conditions. Thus, we wondered whether these proteins were also important for the ability of LitR to inhibit pellicle formation. To evaluate this possibility, we used both epistasis and suppression analyses. Qrr1 is the upstream negative regulator of LitR (5) (Fig. 1), and LitR is epistatic to Qrr1 for pellicle formation (18). This information allowed us to assess whether LitR is in the same pathway as PdeV and VF_A1016 using the ∆*qrr1* mutant in epistasis analyses. In the pellicle assay, loss of *qrr1* was epistatic to loss of both *pdeV* and *VF_A1016* in both double and triple mutants (Fig. S4A and B), suggesting that deletion of *pdeV* and *VF_A1016* is not sufficient to restore biofilm formation to the ∆*qrr1* mutant because (i) Qrr1 controls another unknown component in these conditions or (ii) LitR controls factors other than PdeV and VF_A1016 levels to inhibit static biofilm formation.

**Fig 7:**
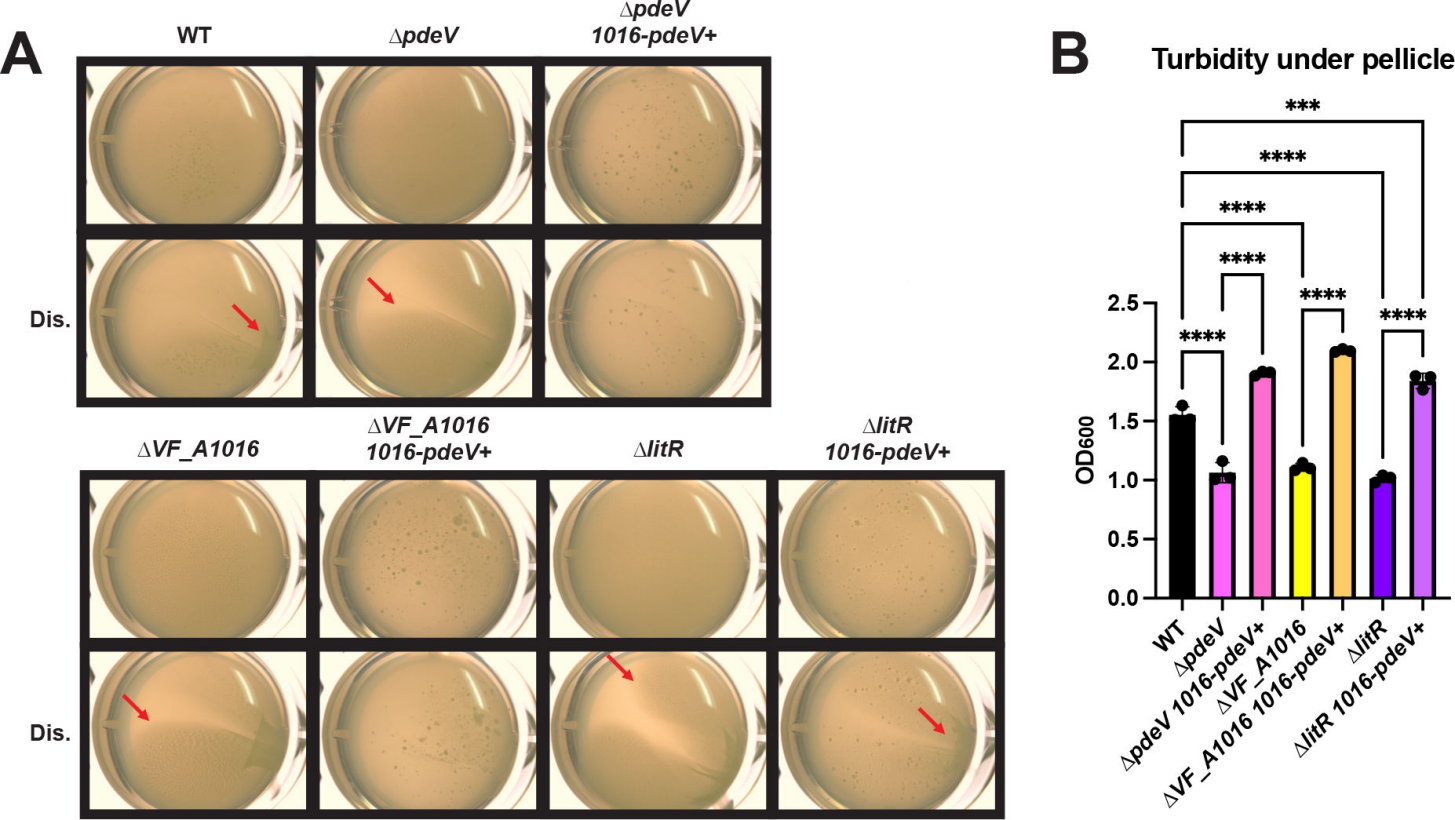
Co-expression of *VF_A1016* and *pdeV* suppresses pellicle formation by the ∆*litR* mutant. (**A**) WT (ES114), the ∆*pdeV* mutant (KV8969), the ∆*pdeV* mutant expressing P*nrdR-VF_A1016*-P*nrdR-RBS-pdeV-flag* (*1016-pdeV*^+^) (BF538), the *VF_A1016* mutant (BF48), the ∆*VF_A1016 1016-pdeV*^+^ strain (BF539), the ∆*litR* mutant (KV10494), and the ∆*litR 1016-pdeV*^+^ strain (BF543) were assessed after 72 h of static growth at 24°C in LBS + 10 mM CaCl_2_. Images were taken using the Zeiss Stemi 2000-c microscope at 6.5× magnification with and without disruption (Dis.) using a toothpick to indicate stickiness. Red arrows are used to highlight an area of each disrupted pellicle where stickiness and/or cohesiveness is observed. (**B**) Turbidity of the liquid underneath the pellicle was measured by OD_600_ and plotted. Statistics were performed using a one-way ANOVA corrected for multiple comparisons by Tukey’s test; ***P-value: 0.0001, ****P-value < 0.0001.

We further investigated the contributions of these genes relative to loss of LitR under static conditions by introducing an additional copy of *VF_A1016* and/or *pdeV* at a non-native site in the chromosome. The addition of a second copy of either *VF_A1016* or *pdeV* to the Δ*litR* mutant resulted in ∆*litR* mutant levels of stickiness and only a small, but reproducible increase in turbidity (Fig. S4C-F). In contrast, the insertion of both *VF_A1016* and *pdeV* controlled by P*nrdR* restored a WT-like biofilm to the Δ*litR* mutant (Fig. 7), suggesting that the ∆*litR* mutant phenotype could be overcome by a second copy of both *VF_A1016* and *pdeV*. These data indicate that multiple factors contribute to biofilm inhibition under static conditions.

### LitR binds to confirmed and putative biofilm regulators/components

**Fig 8:**
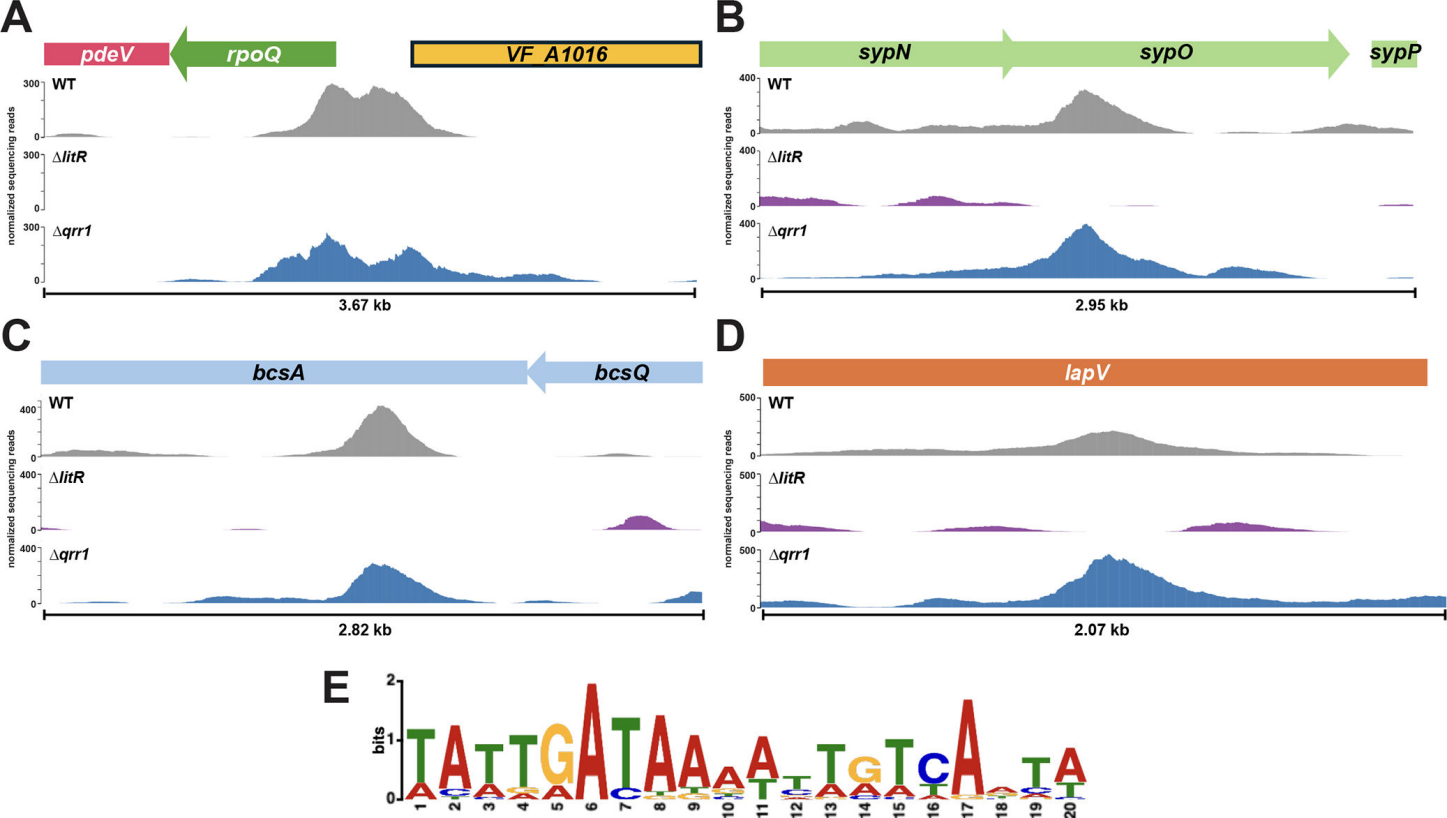
ChIP-seq analysis reveals LitR-binding sites for *rpoQ*, *VF_A1016*, and other biofilm-relevant genes. (**A–D**) DNA bound to LitR from WT (ES114), the ∆*litR* mutant (KV10494), and the ∆*qrr1* mutant (TIM305) was sequenced from samples grown with shaking in LBS + 10 mM CaCl_2_ at 24°C to an OD_600_ of 1.0. The resulting peaks for (**A**) the *rpoQ* and *VF_A1016* regulatory region, (**B**) within *sypO*, (**C**) within *bcsA*, and (**D**) within *lapV* are shown using Jbrowse software (40). (**E**) The top 20 signal values from the WT vs ∆*litR* mutant comparison, as well as the peak within the *rpoQ*/*VF_A1016* intergenic region, were analyzed by MEME (41) to determine the LitR binding motif.

Our *E. coli* experiments suggested that LitR directly controls *rpoQ-pdeV* and *VF_A1016* transcription (Fig. S1C and E). To determine whether the effect could be direct in *V. fischeri*, we performed a ChIP-seq experiment to map LitR-binding sites using WT, the ∆*qrr1* mutant, and the ∆*litR* mutant grown in LBS containing 10 mM calcium chloride with shaking. The antibody used for ChIP-seq (anti-LuxR*^Vca^*) is specific for LuxR of *Vibrio campbellii* strain ATCC BAA-1116 (38), an isolate that was originally classified as *V. harveyi* (39). This antibody successfully and specifically bound *V. fischeri* LitR (Fig. S5). Using this approach, we observed LitR binding in the intergenic region between *rpoQ* and *VF_A1016*, demonstrating that LitR directly binds to that region to potentially control gene expression (Fig. 8A). We also observed binding within various known biofilm-relevant genes such as *sypO*, *bcsA*, and *lapV* (Fig. 8B-D). However, instead of binding to the noncoding regions of these biofilm-regulating genes, LitR bound to the coding sequences of these genes. These data implicate LitR in directly binding to inter- or intragenic regions of biofilm-relevant genes.

In addition to the genes listed, some c-di-GMP modulating enzyme encoding genes, *VF_A0152*, *VF_A0244*, and *VF_A0551*, were identified, as well as genes that LitR is known or hypothesized to regulate, such as *ainS*, *VF_1200*, and *dns* (Table S1) (6, 11, 13, 42). In addition, genes previously identified as differentially regulated in a ∆*luxO* mutant vs a phosphomimetic LuxO variant (upregulated LitR vs downregulated LitR) in a microarray analysis were also identified in the ChIP-seq, namely *fliL*, *fliE*, *flaD*, *flaF*, and *tfoY* (7) (Table S1).

Using the most significant peaks in the data set, we extrapolated a consensus binding sequence for LitR using MEME analysis (41) (Fig. 8E). This sequence is similar to the motifs found for LuxR in *V. campbellii*, SmcR in *Vibrio vulnificus*, and LitR in *V. fischeri* isolate FQ-A001 (12, 43, 44), suggesting that the binding site is highly conserved. Taken together, LitR appears to directly interact with multiple biofilm-related genes, potentially resulting in LitR-dependent biofilm regulation. For *rpoQ* and *VF_A1016*, this interaction seems to be, at least partially, through direct interactions between LitR and the *rpoQ-pdeV*/*VF_A1016* regulatory region.

### LitR controls the transcription of genes identified by ChIP-seq

Finally, we sought to determine whether some of the genes bound by LitR, as detected in the ChIP-seq, could be transcriptionally controlled by LitR, and if so, whether LitR activates or represses. LitR affects a variety of phenotypes, including biofilm formation, type VI secretion, motility, the acetate switch, and luminescence (4, 6, 7, 9–12, 18). We thus assessed the impact of loss of LitR on transcription of three genes (*tfoY*, *VF_1200*, and *aceB*), involved in some of those phenotypes, using promoter-*lacZ* reporter fusions and β-galactosidase activity assays.

**Fig 9:**
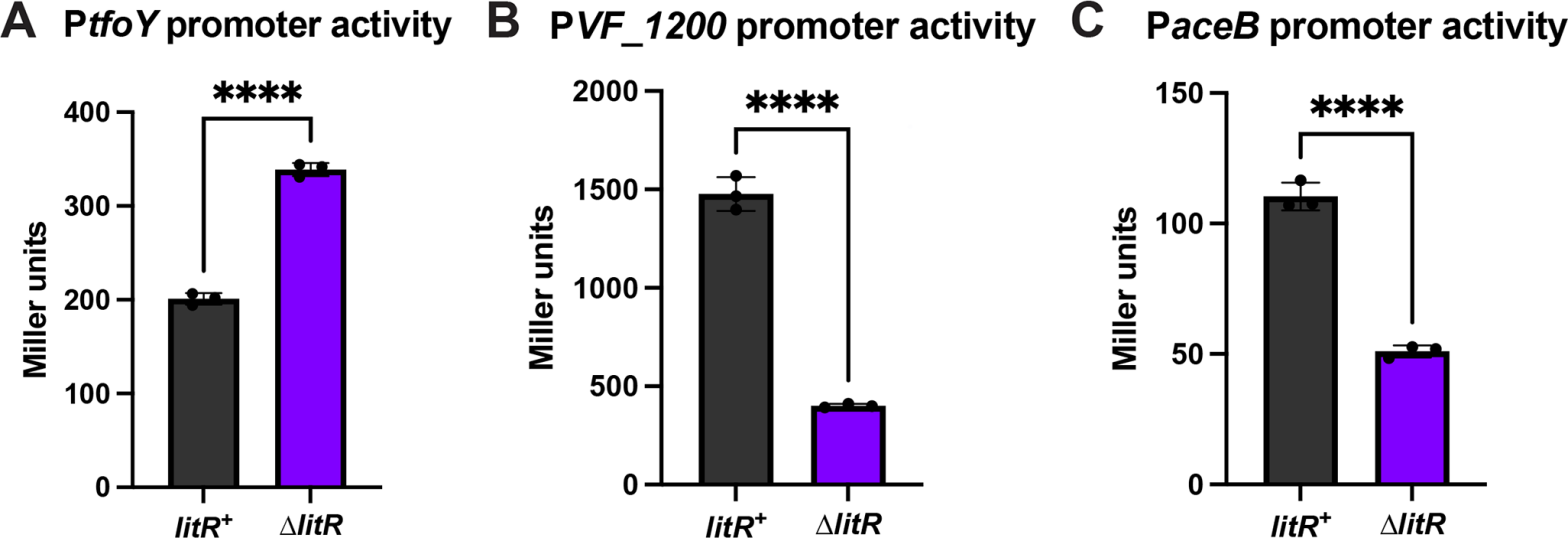
LitR regulates selected genes identified with ChIP-seq. The *litR*^+^ strain and the ∆*litR* mutant, both carrying a ∆*sypQ* mutation and the (**A**) P*tfoY-lacZ* reporter (KV10945 and KV10960), (**B**) P*VF1200-lacZ* reporter (BF677 and BF678), or (**C**) P*aceB-lacZ* reporter (KV10954 and KV10957) were assessed after 22 h of shaking growth in LBS with 10 mM CaCl_2_ at 24°C. Cell extracts were tested for β-galactosidase activity as a measure of promoter activity. Statistics were performed using an unpaired t-test; ****P-value < 0.0001.

In *V. cholerae*, TfoY induces the expression of type VI secretion system genes, promoting cellular defense mechanisms (45). It also controls motility (45, 46). While the former connection to type VI has yet to be established for *V. fischeri*, loss of TfoY diminishes motility (6). Analysis of a *tfoY* promoter-*lacZ* fusion indicated that LitR inhibits *tfoY* transcription; β-galactosidase activity was increased in the Δ*litR* mutant relative to the *litR*^+^ control (Fig. 9A). Introduction of the *litR* plasmid pPMF5 caused a decrease in β-galactosidase activity relative to the vector control, indicating complementation (Fig. S6A). We also mutated the predicted LitR-binding site of *tfoY* based on the ChIP data, which resulted in similar levels of promoter activity as the ∆*litR* mutant (Fig. S6A). These data are consistent with the known inhibition of motility by LitR (7, 10).

The putative diguanylate cyclase VF_1200 inhibits motility in both ES114 and strain KB2B1 (11, 47). Previously, it was speculated that LitR could promote the transcription of *VF_1200* to inhibit motility (11). Indeed, LitR induces *VF_1200* expression (Fig. 9B; Fig. S6B). As with the *tfoY* regulatory region, we also mutated the predicted LitR-binding site of the *VF_1200* regulatory region and found that there was no significant difference in P*VF_1200* promoter activity between the ∆*litR* mutant and the strain in which the binding site was mutated (Fig. S6B). These data suggest that the negative impact on motility by LitR could also be exerted by increasing c-di-GMP levels via VF_1200.

LitR has been implicated in controlling acetate uptake (9), and *aceB*, the most significant peak in the ChIP-seq data set, is known to be important when acetate is the sole carbon source in *E. coli* (28, 29). Therefore, we also examined whether LitR regulates *aceB* expression. Our β-galactosidase assay results show that the ∆*litR* mutant had decreased *aceB* expression, and introduction of the *litR*-expression plasmid increased expression (Fig. 9C; Fig. S6C). Mutation of the predicted LitR binding site of *aceB* resulted in slightly increased levels of P*aceB* promoter activity compared to the ∆*litR* mutant (Fig. S6C), which could suggest that the mutations may not have completely abolished LitR binding to P*aceB*. However, in totality, these data indicate that LitR positively controls *aceB* transcription.

Thus, we have validated three of the potentially affected target genes, as well as *VF_A1016* and *rpoQ* (*pdeV*), as being controlled by LitR, suggesting that additional ChIP-seq-identified binding sites may also represent important sequences mediating transcriptional control by LitR. Taken together, our data indicate that LitR exerts transcriptional control over a substantial number of genes, explaining its contributions to a large number of phenotypes in *V. fischeri*.

## DISCUSSION

Our phenotypic and β-galactosidase assays demonstrated that the transcription factor LitR promotes the expression of *pdeV* and *VF_A1016*, which, in turn, inhibit *V. fischeri* biofilms. In addition, ChIP-seq data revealed not only that LitR directly interacted with the *pdeV* and *VF_A1016* regulatory region, but that LitR bound to many other sites within the *V. fischeri* chromosome to, presumably, regulate a variety of processes. These data further our knowledge of the effects of LitR and quorum sensing on *V. fischeri* physiology, including biofilm formation.

We note that many of the LitR-dependent transcriptional effects we observed were quite minor, with the loss of *litR* leading to fold changes in Miller units in the range of only 1.4–1.6 for several promoter-*lacZ* fusions. It is not possible to conclude that the impact of LitR on any individual gene affects biofilm formation. Rather, LitR-mediated transcriptional control over multiple biofilm-relevant genes, as we have observed, may be necessary to exert a substantial effect on *V. fischeri* biofilms. Alternatively, the control over these genes by LitR may contribute only a minor impact on biofilm formation and, instead, LitR may inhibit biofilms through a different pathway entirely, such as by modulating acetate metabolism via *acs* and *aceB* (9).

We have previously noted that growth conditions (e.g., static vs. shaking) alter the relative importance of known biofilm factors for *V. fischeri* (18, 20, 48, 49). For example, biofilms formed by the ∆*litR* mutant only depend on SYP under static, but not shaking conditions (18). Here, we observed that PdeV and VF_A1016 seemed less important under static growth conditions. Further research is required to understand the relative contributions of biofilm factors under different growth conditions.

Unlike LitR, VF_A1016 is not a transcription factor, so it must indirectly control *bcs* transcription. The cognate response regulator is unknown, but it is predicted to be VF_A1017 (7), which contains a DNA-binding domain. Thus, VF_A1016 may work through VF_A1017 (or a distinct response regulator) to control *bcsQ* transcription directly. Alternatively, VF_A1016 or this two-component pair may indirectly impact cellulose production by, for example, controlling the production of c-di-GMP, the levels of which are important for *bcsQ* transcription and, likely, for cellulose synthesis (19, 24, 50, 51).

When assessing potential LitR-binding sites by ChIP-seq, we identified two distinct peaks in the *rpoQ*/*VF_A1016* region (Fig. 8A) and within other intergenic regions in the data set (Table S1). These dual peaks are reminiscent of those found in *V. harveyi* LuxR ChIP-seq data (52). There, the authors determined that many of the dual peaks are bound not only by LuxR but also by the global DNA-binding protein H-NS. Indeed, these H-NS peaks had higher sequencing reads when *luxR* was mutated in the genome. The authors speculate that these dual peaks are caused by binding of H-NS filaments to two nearby regions of DNA. If this phenomenon occurs in *V. fischeri*, LitR might displace H-NS at these locations, leading to the dual peaks we see at the *rpoQ*/*VF_A1016* intergenic region as well as those around *VF_A0367*/*VF_A0368* and *hns*/*VF_1630* (Table S1), the latter of which implicates LitR in binding to and affecting *hns* transcription. More studies would need to be done to determine whether the LuxR/H-NS interactions found in *V. harveyi* hold true in *V. fischeri*.

In the data set, peaks were identified within many open reading frames (Table S1), including in *sypO*, *bcsA*, and *lapV* (Fig. 8B through D). ChIP-seq peaks have been discovered within coding regions before (e.g., [44, 53]) and were hypothesized to (i) coordinately work with binding sites in nearby promoters to influence transcription, (ii) function as binding sites to regulate adjacent genes, or (iii) regulate an intragenic sRNA (54). However, there were no peaks present in the nearest regulatory regions to these genes in our data set, indicating that the first hypothesis is unlikely to hold true. For the second hypothesis, there were at least 1,300 bp present between *sypO*, *bcsA,* and their respective downstream gene. In addition, the gene downstream of *lapV* is encoded in the opposite direction. Thus, it seems unlikely that LitR binding within *sypO*, *bcsA*, or *lapV* would affect *sypP*, *bcsB*, or *VF_1505* transcription, respectively. Lastly, experiments identifying possible intragenic sRNAs have not been performed in *V. fischeri*. Therefore, more studies are needed to determine if the third hypothesis is true. It is, however, possible that binding at that location could cause steric hindrance of transcription and therefore, inhibit protein production. This phenomenon could provide an explanation for why pellicles formed by the ∆*litR* mutant were dependent on SYP, but loss of LitR did not affect *sypA* transcription (18). Future studies should assess the consequences, if any, of LitR binding in this position in the genome.

The ChIP-seq results not only provided information about biofilm-relevant genes but also identified many other genes with LitR-binding sites, such as those encoding sensor kinases, transcriptional regulators, chemotaxis-related genes, and transporters (Table S1). Some of these genes were previously examined for LitR transcriptional control. For example, genes encoding the flagellin gene *flaF* and the methyl-accepting chemotaxis proteins VF_1133 and VF_2042 were shown to be controlled by LitR by RT-qPCR analysis (55). Another gene of interest, *gfcE*, whose protein product is implicated in exopolysaccharide export, was upregulated by LitR in unpublished microarray analyses (55) (Schaefer and Ruby, unpublished; Studer et al., unpublished). These data support the notion that the ChIP-seq identified genes are likely transcriptionally controlled by LitR. Of note, the microarray and RT-qPCR analyses were performed in SWT, suggesting that, despite the difference in media, LitR may control transcription of these genes in both SWT and LBS + calcium conditions.

Of all the peaks in the ChIP-seq data set, the most striking was found in the intergenic region of *aceB* (Table S1), which encodes malate synthase (29). In *E. coli, aceB*, *aceA*, and *aceK* are transcriptionally activated in conditions where acetate is the sole carbon source (28, 29). When active, these three proteins prevent the loss of usable carbons by diverting isocitrate from the Krebs cycle. LitR had previously been shown to induce *acs* transcription, whose protein is important for acetate uptake (9). We verified that LitR activates *aceB* (Fig. 9C; Fig. S6C), though it is still unknown if LitR-dependent activation of *aceB* is a result of LitR binding to the *aceB* regulatory region. We speculate that, perhaps, LitR induces *aceB*, *aceA*, and *acs* transcription to coordinately restrict acetate to the cytoplasm and prevent the acidification of the surrounding media.

Given that LitR was first identified as a positive regulator of the gene for LuxR, the proximal regulator of the *lux* (luminescence) operon (4, 56, 57), it is worth noting that our ChIP-seq did not identify *luxR* or the divergently transcribed *lux* operon as a target (Table S1). However, a previous study determined that LitR bound to the *luxR* regulatory region by a gel shift (4). There is a possibility that the discrepancy is because the *in vitro* conditions used for the gel shift did not sufficiently recapitulate the environment in the cell, leading to visualization of binding that was not identified by ChIP-seq. Alternatively, LitR may only be important for *luxR* transcription early in the bioluminescence regulatory cascade, becoming unnecessary once LuxR is produced. If so, then perhaps the time point we chose for our assay, an OD_600_ of 1.0, is past the optimal window for LitR-P*luxR* binding.

Taken together, LitR-dependent regulation of biofilm formation is complex. In addition to SYP and cellulose (18), we determined that LapV is important for ∆*litR* mutant biofilms. LitR may indirectly control LapV and cellulose through its direct interaction with the *rpoQ* (*pdeV*) and *VF_A1016* intergenic region. LitR could also contribute to biofilm formation by binding and impacting transcription of any of a variety of other genes identified by the ChIP-seq. In addition, the putative LitR binding site we identified matched that of the *clpV-hcp* intergenic region of another *V. fischeri* isolate, FQ-A001 (12). Although that region, and other associated genes involved in the type VI secretion system 2, are absent in strain ES114, it appears that there is sufficient conservation in the putative LitR-binding site that the genes identified by ChIP-seq in ES114 can likely be used to inform on LitR regulation in FQ-A001 and other isolates. Overall, this work has expanded our knowledge of not just LitR-dependent biofilm regulation, but of the *V. fischeri* LitR regulon as a whole. Moreover, our ChIP data provide numerous additional avenues for research on LitR-mediated transcriptional control over *V. fischeri* physiology.

## MATERIALS AND METHODS

### Strain construction and media

*V. fischeri* was maintained and strains were constructed as previously described (58, 59). Briefly, *V. fischeri* strains (Table 1; Table S2) were grown overnight at 28°C in LBS (10 g/L tryptone, 5 g/L yeast extract, 20 g/L NaCl, 50 mM Tris pH 7.5) (59) or tTBS (10 g/L tryptone, 20 g/L NaCl, and 50 mM Tris pH 7.5) (48). Agar was added at 15 g/L if needed. *E. coli* was grown in LB (10 g/L tryptone, 5 g/L yeast extract, and 10 g/L NaCl) at 37°C with the addition of 0.3 mM thymidine for the auxotrophic π3813 strain (60). Plasmids used in this study are shown in Table 2. Antibiotic concentrations for *V. fischeri* were: chloramphenicol (Cm, 1 µg/mL), kanamycin (Kan, 100 µg/mL), erythromycin (Erm, 2.5 µg/mL), spectinomycin (Spec, 200 µg/mL), and trimethoprim (Trim, 10 µg/mL). Antibiotic concentrations for *E. coli* were as follows: Cm (12.5 µg/mL), Kan (50 µg/mL), and ampicillin (Amp, 100 µg/mL). DNA transformations were performed using a defined Tris-minimal medium (58). Genomic DNA (gDNA) or DNA that was amplified by PCR and splicing by overlap extension (61) was inserted into the *V. fischeri* chromosome by *tfoX*-mediated transformation (6, 30), and recombinants were selected using antibiotics. In certain cases, a non-native promoter, P*nrdR* (36), was used in place of the native promoter. The primers that were utilized are in Table 3 and define the end points of the fragments utilized for transformation. The antibiotic cassettes were removed by tri-parental conjugation with an *E. coli* strain carrying the flippase enzyme on plasmid pKV496 (58, 62).

**TABLE 1:** Strains used in this study

Strains	Genotype^*a*^	Construction	Reference
ES114	Wild type	N/A	(63)
BF11	*litR*::Kan^r^ ∆*bcsA*::FRT-Trim^r^	N/A	(18)
BF13	*litR*::Kan^r^ ∆*sypQ*::FRT-Erm^r^	N/A	(18)
BF48	∆*VFA1016*::FRT	Erm^r^ removed from KV8026	This study
BF49	∆*rpoQ*::FRT	Spec^r^ removed from KV9339	This study
BF76	∆*lapV-1500*::FRT *litR*::Erm^r^	TT KV8629 with gJB19	This study
BF84	∆*VFA1016*::FRT ∆*sypQ*::FRT-Erm^r^	TT BF48 with gKV8191	This study
BF85	∆*VFA1016*::FRT *bcsA*::Tn5	TT BF48 with gKV8408	This study
BF89	∆*VFA1016*::FRT-Erm^r^ ∆*lapV-1500*::FRT	TT KV8629 with KV8026	This study
BF91	∆*litR*::FRT-Spec^r^ IG (Erm^r^)::P*nrdR-RBS-pdeV-flag*	TT KV9482 with gKV9740	This study
BF109	∆*sypQ*::FRT-Cm^r^ ∆*bcsA*::FRT-Trim^r^ ∆*VFA1016*::FRT-Erm^r^	TT KV8753 with gKV8026	This study
BF203	∆*VFA1016*::FRT IG (Erm^r^)::P*nrdR-VFA1016*	TT BF48 with gKV10111	This study
BF204	∆*VFA1016*::FRT ∆*litR*::FRT-Spec^r^	TT BF48 with gKV9740	This study
BF212	∆*litR*::FRT IG (Erm^r^)::P*nrdR-VFA1016*	TT KV10494 with gKV10111	This study
BF237	IG::P*sypA-lacZ* ∆*sypQ*::FRT	N/A	(18)
BF244	IG::P*sypA-lacZ* ∆*sypQ*::FRT ∆*VFA1016*::FRT-Erm^r^	TT BF237 with gKV8026	This study
BF255	IG::P*bcsQ-lacZ* ∆*sypQ*::FRT	N/A	(18)
BF259	IG::P*bcsQ-lacZ* ∆*sypQ*::FRT ∆*VFA1016*::FRT-Erm^r^	TT BF255 with gKV8026	This study
BF297	IG::P*rpoQ-lacZ* ∆*sypQ*::FRT	Erm^r^ removed from BF268	This study
BF298	IG::P*rpoQ-lacZ* ∆*sypQ*::FRT ∆*litR*::FRT	Spec^r^ and Erm^r^ from BF285	This study
BF299	IG::P*VFA1016-lacZ* ∆*sypQ*::FRT	Erm^r^ removed from BF291	This study
BF310	IG:: P*VFA1016-lacZ* ∆*sypQ*::FRT ∆*litR*::FRT-Spec^r^	TT BF299 with gKV9740	This study
BF538	∆*pdeV*::FRT IG (Erm^r^)::P*nrdR-VFA1016*-P*nrdR-RBS-pdeV-flag*	TT KV8969 with gBF529	This study
BF539	∆*VFA1016*::FRT IG (Erm^r^)::P*nrdR-VFA1016*-P*nrdR-RBS-pdeV-flag*	TT BF48 with gBF529	This study
BF543	∆*litR*::FRT IG (Erm^r^)::P*nrdR-VFA1016*-P*nrdR-RBS-pdeV-flag*	TT KV10494 with gBF529	This study
BF598	IG::P*lapV-lacZ* ∆*sypQ*::FRT	Erm^r^ removed from BF594	This study
BF599	∆*litR*::FRT-Spec^r^ IG::P*lapV-lacZ* ∆*sypQ*::FRT	Spec^r^ and Erm^r^ removed from BF597	This study
BF607	∆*pdeV*::FRT ∆*litR*::FRT-Spec^r^	TT KV8969 with gKV9740	This study
BF609	IG::P*lapV-lacZ* ∆*sypQ*::FRT ∆*VFA1016*::FRT-Spec^r^	TT BF598 with gKV10619	This study
BF646	∆*qrr1* ∆*VFA1016*::FRT-Erm^r^	TT TIM305 with gKV8026	This study
BF647	∆*qrr1* ∆*pdeV*::FRT-Spec^r^	TT TIM305 with gKV9324	This study
BF666	∆*qrr1* ∆*VF_A1016*::FRT-Erm^r^ ∆*pdeV*::FRT-Spec^r^	TT BF646 with PCR product amplified with primers 2618 & 2621 (KV9324)	This study
BF669	∆*bcsA*::FRT ∆*lapV-1500*::FRT ∆*litR*::FRT-Spec^r^	TT BF665 with gKV9740	This study
BF677	IG::P*VF1200-lacZ ∆sypQ*::FRT-Erm^r^	TT BF279 with gKV8191	This study
BF678	IG::P*VF1200-lacZ ∆sypQ*::FRT-Erm^r^ ∆*litR*::FRT	TT BF281 with gKV8191	This study
KV8969	∆*pdeV*::FRT	N/A	(23)
KV9410	∆*pdeV*::FRT ∆*sypQ*::FRT-Cm^r^	N/A	(23)
KV9895	∆*sypQ*::FRT	N/A	(18)
KV9918	∆*pdeV*::FRT-Spec^r^ IG (Erm^r^)::P*nrdR-RBS-pdeV-flag*	TT KV9482 with gKV9324	This study
KV10494	∆*litR*::FRT	N/A	(18)
KV10670	∆*sypQ*::FRT IG::P*rpoQ*-T1-*lacZ*	Erm^r^ removed from KV10593	This study
KV10775	∆*sypQ*::FRT IG::P*VFA1016*-T1-*lacZ*	Erm^r^ removed from KV10766	This study
KV10803	∆*sypQ*::FRT IG::P*rpoQ*-T2-*lacZ*	Erm^r^ removed from KV10797	This study
KV10804	∆*sypQ*::FRT IG::P*VFA1016*-T2-*lacZ*	Erm^r^ removed from KV10798	This study
KV10865	∆*sypQ*::FRT IG::P*VFA1016*-T3-*lacZ*	Erm^r^ removed from KV10859	This study
KV10867	∆*sypQ*::FRT IG::P*rpoQ*-T3-*lacZ*	Erm^r^ removed from KV10861	This study
KV10931	∆*rpoQ*::FRT IG (Erm^r^)::P*rpoQ-rpoQ*	TT BF49 with gKV10910	This study
KV10932	∆*rpoQ*::FRT IG (Erm^r^)::P*nrdR-RBS-pdeV-flag*	TT BF49 with gKV9482	This study
KV10945	IG (Erm^r^)::P*tfoY-lacZ* ∆*sypQ*::FRT	TT KV9895 with gDNA from an intermediate strain made by TT KV7371 with SOE product amplified with primers 2185 & 2090 (pKV502) and 4516 & 2951 (ES114), and 2822 & 2876 (KV9466)	This study
KV10954	IG (Erm^r^)::P*aceB-lacZ* ∆*sypQ*::FRT	TT KV9895 with gKV10944	This study
KV10957	IG (Erm^r^)::P*aceB-lacZ* ∆*sypQ*::FRT ∆*litR*::FRT-Spec^r^	TT KV10951 with gKV10944	This study
KV10960	IG (Erm^r^)::P*tfoY-lacZ* ∆*sypQ*::FRT ∆*litR*::FRT-Spec^r^	TT KV10951 with gDNA from an intermediate strain made by TT KV7371 with SOE product amplified with primers 2185 & 2090 (pKV502) and 4516 & 2951 (ES114), and 2822 & 2876 (KV9466)	This study
KV11163	IG (Erm^r^)::P*VF1200-lacZ ∆sypQ*::FRT	TT KV9895 with gKV9758	This study
KV11164	IG (Erm^r^)::P*VF1200-lacZ ∆sypQ*::FRT ∆*litR*::FRT-Spec^r^	TT KV10951 with gKV9758	This study
KV11165	IG (Erm^r^)::P*aceB*-mutated-*lacZ* ∆*sypQ*::FRT	TT KV9895 with gKV11120	This study
KV11166	IG (Erm^r^)::P*aceB*-mutated-*lacZ* ∆*sypQ*::FRT ∆*litR*::FRT-Spec^r^	TT KV10951 with gKV11120	This study
KV11167	IG (Erm^r^)::P*tfoY*-mutated-*lacZ* ∆*sypQ*::FRT	TT KV9895 with gKV11136	This study
KV11168	IG (Erm^r^)::P*tfoY*-mutated-*lacZ* ∆*sypQ*::FRT ∆*litR*::FRT-Spec^r^	TT KV10951 with gKV11136	This study
KV11169	IG (Erm^r^)::P*VF1200*-mutated-*lacZ ∆sypQ*::FRT	TT KV9895 with gKV11137	This study
KV11170	IG (Erm^r^)::P*VF1200*-mutated-*lacZ ∆sypQ*::FRT ∆*litR*::FRT-Spec^r^	TT KV10951 with gKV11137	This study
TIM305	∆*qrr1*	N/A	(5)

^*a*^ N/A, not applicable; IG, gene inserted at intergenic region between genes *yeiR* and *glmS* along with an FRT scar, with one exception: KV7371 does not contain an FRT scar; IG (Erm), gene inserted between *yeiR* and *glmS* along with FRT-Erm^r^; TT, TfoX-mediated transformation using *tfoX*-overexpressing version of indicated strain; trunc, truncation; RBS, idealized ribosome-binding site; FLAG, FLAG-epitope tagged; FRT, Flippase Recognition Target; if not followed by an antibiotic resistance gene, then the antibiotic cassette was flipped out leaving an FRT scar within the chromosome.

**TABLE 2:** Plasmids used in this study

Name	Description	Reference
pEVS104	Conjugal helper plasmid (Kan^r^)	(64)
pJJC4	*tfoX^+^ +* Cm^r^	(6)
pJET-P*rpoQ-lacZ*	pJET + SOE product amplified with primers 975 & 974 (BF298) (Amp^r^)	This study
pJET-P*VFA1016-lacZ*	pJET + SOE product amplified with primers 975 & 974 (BF310) (Amp^r^)	This study
pKV494	pJET + FRT-Erm^r^	(62)
pKV496	*flp*^+^ + Kan^r^	(62)
pKV502	pJET + *yeiR*-FRT-Erm^r^	(62)
pKV503	pJET + *glmS*	(62)
pKV506	pJET + *yeiR*-FRT-Erm^r^-P*nrdR*	(62)
pKV521	pJET + Spec^r^	(62)
pLosTfoX	*tfoX* ^+^	(30)
pLosTfoX-Kan	*pLosTfoX* + Kan^r^	(36)
pPMF5	pVSV105 + *litR* (Cm^r^)	(6)
pVSV105	Cm^r^	(65)

**TABLE 3:** Primers used in this study

Primer number	Sequence^*a*^
423	GGTTGACAGGTTTCTTGGCG
974	GCTAAAGCGGTGACGGTGGAGTAG
975	CCTCACCCCAGATGGTTTGGCA
1487	GGTCGTGGGGAGTTTTATCC
1821	tcctgtgtgaTGAGCTGACTAATAAAAGTATTAG
1965	ATCAGTCTGGTTTCTTTTTCG
1966	TACTTCTGATGTGGTTTGTTG
2022	gcgaggctggccggcgtcgacAGGCCAACTCCTCTTCTGC
2025	cagacaattgacggctctagaGAGGAGGTTTGAGTAAGTTTC
2089	CCATACTTAGTGCGGCCGCCTA
2090	CCATGGCCTTCTAGGCCTATCC
2097	CCATACTTAGTGCGGCCGCCTAGAAGTTCCTATTCTCTAG AAAGTATAGGAACTTCGGATCCTAAGAGTGTGTTGATAGTGCAG
2122	taggcggccgcactaagtatggTAGTAGCGTCATAAACAATCCT
2123	ggataggcctagaaggccatggTAAGGACGATGCATGACTTCG
2185	CTTGATTTATACAGCGAAGGAG
2196	TCCATACTTAGTGCGGCCGCCTA
2224	TCGCTTGCTTCTACTTCTTTAC
2225	taggcggccgcactaagtatggGTAACCACCTAAAATCCATTTAAC
2226	ggataggcctagaaggccatggAATGACCTTCCAGAAATCACTG
2227	TGAAATCGCTTGAGTATCTGTAAG
2290	AAGAAACCGATACCGTTTACG
2354	gattataaagatgatgatgataaataaCCATACTTAGTGCGGCCGCCTA
2618	AGAGCAGCTCGTGAGTTATC
2619	taggcggccgcactaagtatggAACGCCATCATCCATATAAATACAAC
2620	ggataggcctagaaggccatggGCTATTGCTTAGTATGACTGCTC
2621	CTTCCATAGTAAGAACCTCTGC
2808	taggcggccgcactaagtatggATTTGATTCGAATTGATGTAGTTC
2809	ggataggcctagaaggccatggGTTAATAAAACAACGCATTAATTAG
2822	AGGAAACAGCTATGACCATGATTACGGATTCAC
2843	ACAATCCTTGTTCATAAAGGCC
2844	taggcggccgcactaagtatggACCCTGTTCTTCGCAGACTC
2845	ggataggcctagaaggccatggAGCACTGGGTTGATATTGTTTC
2846	CCGATGAATACTCAATTAGTGTTC
2871	ggataggcctagaaggccatggTATGTTGAGGTTGTATTTATATGG
2872	taggcggccgcactaagtatggCTAAGCAATAGCTGACATTTTTTCCC
2876	GAAACGCCGAGTTAACGCC
2877	ggataggcctagaaggccatggGACAATGTCTCTTTTAGGATAAAC
2878	catggtcatagctgtttcctCCTTCTATATTTTTGATTTCTAC
2892	ttatttatcatcatcatctttataatcAGCAATAGCTGACATTTTTTCCC
2913	GGTGGATTCGAGAATCCATTG
2951	ggataggcctagaaggccatggGTAGTAGTGTTTCAAACCGCC
2983	ggataggcctagaaggccatggCAAACATATATAAACGCCAAAG
2984	catggtcatagctgtttcctATTTTTAGTCTAATTACAAGAG
3025	taggcggccgcactaagtatggCGAAGTCATGCATCGTCC
3078	ggataggcctagaaggccatggTGTACCTAGCTGTTTTATATCTC
3080	ggataggcctagaaggccatggTGTTCATAAAGGCCTAAGTGC
3233	taggcggccgcactaagtatggaTTATTTTATTGTCATGATTTTTTCTAA
3243	GAACTCACTGTTTTTTCTAGTG
3265	ggataggcctagaaggccatggAGGATTGTTTATGACGCTACTAGATC
3276	catggtcatagctgttTCCTTGTTCATAAAGGCCTAAGTGC
3277	catggtcatagctgttTCCTTGTACCTAGCTGTTTTATATCTCTAGC
4328	ggataggcctagaaggccatggATCACAGATTATATTGATAAATATG
4330	ggataggcctagaaggccatggTTAATTTACGGTATTGCCGC
4332	ggataggcctagaaggccatggAAATAACATATTTATCAATATAATC
4438	ggataggcctagaaggccatggACTTGTGATCTTAATAACAAATC
4470	ggataggcctagaaggccatggTTAAAAACAAAAGATAATTACTTGC
4491	ggataggcctagaaggccatggCCTATGCTTTACTTATAAGTTGG
4497	GGAGATATTGATATGAACTTGAAAG
4498	CGTTCTTTAATCGAATAATATCTG
4499	GTTACAAAGCGAAAAAACTG
4500	GAATAAAAGTATATAAGTTAATATTCTC
4501	CATTATTCTTAACAAGATCATAAATTCC
4502	CATCATCAATCGCTATGG
4503	CTAAGTTATTTTGAGATAAAGAACTATC
4504	CTAATGCTTTATTATTTGATGAAAGTTC
4510	ggataggcctagaaggccatggTAGTGAATTTCTTGATACAAGGCG
4511	catggtcatagctgtttcctGCGCCTCCGGCAAAATTACATTTG
4516	catggtcatagctgtttcctTGCAATAATTCTCGGTTAGTAAGTC
4540	CAAACTTTTGcccccGATTGCTATATCATCAATC
4541	GCAATCgggggCAAAAGTTTGAACGGAAAAATAC
4544	GACAATTATATCccccGAGCATTTACTCATAATTC
4545	GTAAATGCTCggggGATATAATTGTCAAAATTTAATT
4546	GTAATAAACCTccccGATAGATTTGCGGATAATAATTC
4547	CAAATCTATCggggAGGTTTATTACTGATAATAAAAAGC

^*a*^ Lowercase letters represent tail sequences.

### Pellicle assay

Strains were grown overnight at 24°C in LBS. Following this, the cultures were normalized to an OD_600_ of 0.02 in 2 mL of LBS supplemented with 10 mM CaCl_2_ in 24-well plates. Each strain was inoculated into three individual wells per experiment. The plates were incubated at 24°C for 72 h. At this time point, the pellicles were imaged with and without disruption using a toothpick to assess for stickiness of the pellicle (22, 23). Images were taken using the Zeiss Stemi 2000-c microscope at a magnification of 6.5×. The turbidity of the liquid underneath the pellicle was measured by OD_600_ (18).

### Shaking liquid biofilm assay

After growth in tTBS overnight at 28°C, the strains were normalized to an OD_600_ of 0.05 in 2 mL of tTBS + 10 mM CaCl_2_ in 13 × 100 mm test tubes in triplicate. These cultures were incubated at 24°C for 24 h and subsequently imaged using a phone camera. The turbidity of the liquid, avoiding the biofilm structures, was measured by OD_600_ (20).

### *V. fischeri* β-galactosidase assay

All *V. fischeri* strains assessed by this assay included a ∆*sypQ* mutation to abolish sticky biofilm formation, permitting more accurate OD_600_ measurements. Strains were grown in LBS overnight at 24°C and subcultured 1:100 in 20 mL or 5 mL LBS + 10 mM CaCl_2_ the following day for the mutation or complementation experiments, respectively. Cm was included in the overnight and subculture for plasmid maintenance for the complementation experiments. These cultures were incubated at 24°C with shaking for 22 h. At this time point, 2 mL or 1 mL of the culture for the deletion or complementation/mutated binding site experiments, respectively, was pelleted, and promoter activity of the regulatory regions was examined by the β-galactosidase assay as previously described (20). The OD_420_ and OD_550_ were measured by the Biotek Synergy H1 plate reader and used to calculate the final Miller units (66).

### *E. coli* β-galactosidase assay

The *E. coli* strains carried two plasmids: one plasmid containing a promoter-*lacZ* fusion (Amp) and either pVSV105 (65) or pPMF5 (6) (Cm). To construct the plasmid containing a promoter-*lacZ* fusion, the regulatory region of each gene of interest was amplified by PCR (for primers see Table 3). Each fragment was ligated into a pJET plasmid using the CloneJET PCR cloning kit (ThermoFisher). The plasmid was then introduced by transformation into PIR1 chemically competent *E. coli* cells (Invitrogen). Transformants were selected on LB Amp plates with and without X-gal (5-bromo-4-chloro-3-indolyl-β-D-galactopyranoside) and incubated at 37°C. Blue colonies from the X-gal plates were chosen for further analysis. Plasmid DNA was extracted using the Zymo Research Plasmid Miniprep kit, evaluated by PCR, and confirmed by sequencing, then introduced into MC4100λ *E. coli* (67) made competent using calcium chloride, followed by selection on LB amp plates. Finally, either plasmid pPMF5 or pVSV105 was introduced into the MC4100λ promoter-*lacZ* containing strains by transformation, followed by selection on LB with Amp and Cm.

The resulting strains were grown overnight in 5 mL LB with Amp and Cm at 28°C. The next day, each strain was subcultured to an OD_600_ of 0.05 in 20 mL LB with Amp and Cm and incubated with shaking at 28°C. At 6 hours, 1 mL of the culture was pelleted, and the β-galactosidase assay was performed as described above.

### Reverse transcriptase polymerase chain reaction

WT strains were grown overnight in 5 mL of LBS and subcultured 1:100 in 20 mL of LBS containing 10 mM CaCl_2_ in 125 mL flasks the next day. The cultures were incubated with shaking for 22 h. Subsequently, 10 mL of RNAprotect (Qiagen) was added to 5 mL of culture, and the mixture was centrifuged at 3,000 × *g* for 10 min. RNA was extracted from the samples using the Direct-zol RNA Miniprep Plus Kit (Zymo Research). Contaminating DNA was degraded using RNase-Free DNase (Qiagen). The DNase was removed by reextracting the RNA using the Direct-zol RNA Miniprep Plus Kit. We then synthesized reverse-transcribed DNA (cDNA) for the coding regions within *pdeV* and *rpoQ*, as well as the intergenic region between the two genes, with reagents from the 5′ RACE System for Rapid Amplification of cDNA Ends kit (Invitrogen, version 2.0). The resulting cDNA was cleaned and concentrated with the DNA Clean & Concentrator-5 kit (Zymo Research) and amplified with primers specific for *pdeV*, *rpoQ*, or the intergenic region between the genes with KOD polymerase (Invitrogen). For *pdeV*, cDNA was made using primer 4497, and the cDNA was amplified with primers 4498 and 4499. For the no reverse transcriptase control and the intergenic region between *rpoQ* and *pdeV*, cDNA was made using primer 4500 and the cDNA was amplified with primers 4501 and 4502. For *rpoQ*, cDNA was made using primer 4503, and the cDNA was amplified with primers 4504 and 2913. See Table 3 for primers. The DNA samples were assessed and imaged on a 2% agarose gel.

### Growth curves

*V. fischeri* strains were grown overnight in 5 mL LBS at 24°C. The following day, the strains were normalized to an OD_600_ of 0.05 in 20 mL of LBS + 10 mM CaCl_2_ in 125 mL flasks. The cultures were incubated with shaking at 24°C over a period of 8 h with OD_600_ measurements taken at each hour. Dilutions of the cultures were performed as needed for OD_600_ readings (59).

### Statistics

Each experiment was performed with at least three biological replicates, and representative images are shown. Each supplemental experiment was performed with at least two biological replicates, and representative images are shown. Statistics were done using GraphPad Prism version 10.1.0.

### Western blots

Whole cell lysates were collected from WT, the Δ*litR* mutant, and the ∆*qrr1* mutant after 24, 48, and 72 h and run on an SDS-PAGE gel (4.5%–12%). Proteins were transferred to a 0.45 µM nitrocellulose membrane via wet transfer in transfer buffer (48 mM Tris base, 39 mM glycine, 0.037% SDS, and 20% methanol) at 10 V for 70 min. The membrane was blocked overnight at 4°C in a 5% milk/TBS-T solution (5 g nonfat dry milk per 100 mL buffer, 25 mM Tris-Cl pH 8.0, 125 mM NaCl, and 0.1% Tween 20). The membrane was washed 3 × 5 min rocking in TBS-T at room temperature (R/T) and then treated with a 1:10,000 dilution of anti-RpoB (BioLegend) or a 1:3,000 dilution of anti-LuxR*^Vca^* (38) for 1 h rocking at R/T. After washing the membranes for 3 × 5 min rocking in TBS-T at R/T, the membranes were treated with either a 1:10,000 dilution of anti-mouse IgG, HRP (RpoB; Promega) or a 1:10,000 dilution of anti-rabbit IgG, HRP (LuxR; Promega) for 30 min rocking at R/T. All RpoB primary and secondary antibody dilutions were dissolved in TBS-T, whereas LuxR dilutions were dissolved in 5% milk/TBS-T. The secondary antibodies were washed 3 × 5 min rocking at R/T. To visualize the membranes, Pierce ECL Western Blotting Substrate (Thermo Scientific) was added for 5 min, and images were captured using the autoexposure setting for a chemi blot on Bio-Rad Image Lab software.

### Chromatin immunoprecipitation sequencing

The chromatin immunoprecipitation (ChIP) procedure was modified from a *Bacillus subtilis* procedure (68). Briefly, 50 mL cultures of cells were grown shaking at 24°C in LBS + 10 mM CaCl_2_ until they reached an OD_600_ of 1.0. For the whole-genome sequencing (WGS; input) samples, a 3 mL aliquot was pelleted before crosslinking, and gDNA was extracted using the ThermoFisher GeneJet DNA Purification Kit. The DNA was diluted to 10 ng/µL in 100 µL of elution buffer and sonicated at 4°C twice with a Qsonica Q800R2 Water Bath Sonicator at 20% amplitude with continuous pulses. WGS samples were stored at −20°C until library preparation. The remaining cultures (47 mL) (immunoprecipitation (IP) samples) were crosslinked by adding 1% formaldehyde (Fisher Scientific) and rocked for 30 min at R/T. The reactions were quenched with 0.5 M glycine and rocked for 5 min at R/T. Cultures were pelleted at 4°C, washed 2 times with 1 mL cold 1× TBS, frozen with liquid nitrogen, and stored at −80°C until needed. During lysis, pellets were resuspended in ChIP Solution A (12.5 mM Tris pH 8, 12.5 mM EDTA, 62.5 mM NaCl, 25% sucrose, and 1 mM PMSF) with 1 mg/mL lysozyme for 45 min at 37°C. An equal volume of 2× IP Buffer was added to the solution, followed by 1 mM PMSF and 1× Protease Inhibitor Cocktail (52). Samples were sonicated twice for 20 min at 70% amplitude with 10 s on/off pulses and then centrifuged to clear cell debris. Sonicated lysates were pre-cleared by rotating for 1 h at 4°C using 50 µL washed Protein A Magnetic Sepharose beads (Cytiva #28967062). Pre-cleared lysates were incubated with 4 µL anti-LuxR*^Vca^* (38) and rotated overnight at 4°C. LuxR is the LitR homolog in *V. campbellii* and has 59.5% identity and 77.5% similarity to *V. fischeri* LitR. The anti-LuxR*^Vca^* antibody was shown to be cross-reactive with *V. fischeri* LitR using western blots (Fig. S5), so it was used to pull down LitR during ChIP-seq. The next day, 50 µL of Protein A magnetic beads were added to the lysate-antibody mixtures and rotated for 1 h at 4°C. The resin was washed 3 times with 1× IP Buffer, three times with Wash Buffer, once with 1× TE, and eluted with TES at 65°C for 20 min. Crosslinks were reversed at 65°C overnight and then treated with 0.2 mg/mL RNaseA (NEB #T3018L) and 0.2 mg/mL Proteinase K (NEB #P8107S). DNA was extracted using a phenol:chloroform:isoamyl alcohol (25:24:1) mixture with ethanol precipitation, then purified using AmpureXP beads (Beckman Coulter, Inc.). WGS and IP libraries were prepared for Illumina NextSeq 550 high-throughput paired-end sequencing using the NEBNext Ultra II DNA Library Prep Kit for Illumina (NEB #E7645) and sequenced at the Indiana University Center for Genomics and Bioinformatics (CGB). Three biological replicates were sequenced for each sample.

### ChIP-seq analysis

Illumina paired-end reads were trimmed using fastp (version 0.21.0) with parameters “-l 25 --detect_adapter_for_pe -g -p” (69). Trimmed reads were mapped to the *V. fischeri* ES114 reference genome using bowtie2-2.3.5.1 (70) and were run with the –no-mixed option. There was low-level contamination with *E. coli* DNA, so we filtered out reads with more than one non-matching base. Read pairs with the same end points were filtered to avoid potential PCR duplicates. The input reads had uneven coverage due to ongoing DNA replication in the growing cells, with higher coverage at the origin and reduced coverage at the terminus. The input coverage was estimated and used to normalize the ChIP-seq coverage (for visualization and validation) and to normalize the start frequencies using custom Perl scripts.

Artificial read distributions were then randomly generated from the normalized start frequencies and the corresponding insert sizes for a given read library. The input distribution was then subtracted from the corresponding experimental sample using custom Perl scripts. This resulted in an input-normalized artificial read set for each experimental sample. One set of samples from a particular experimental condition could then be directly compared in MACS (version 2.2.9.1) against another set of experimental samples to identify peaks distinct to one experimental condition or another. The unnormalized data were used to verify the validity of these peaks and to set biologically meaningful q-value thresholds. The workflow is depicted in Fig. S7.

The data set was filtered to only include peaks that were higher in WT or the ∆*qrr1* mutant compared to the ∆*litR* mutant, our negative control. By assessing the peaks using JBrowse (40), we determined the point at which the average raw reads of WT or the ∆*qrr1* mutant were at least fourfold higher than the negative control, which was a MACS assigned signal value of 5. Any peak with a signal value below 5 was deemed not significant. We then used the DNA sequences of the peaks with the 20 highest signal values, as well as the peak within the *rpoQ*/*VF_A1016* intergenic region, and performed a MEME analysis (41) to determine the consensus binding motif. For each peak, we annotated the predicted function of the gene or adjacent genes using Uniprot (71).

## Data Availability

The ChIP-seq data discussed in this publication have been deposited in NCBI’s Gene Expression Omnibus (GEO) (72): GSE288643. The link to the Jbrowse session with the uploaded sequences is: https://jbrowse.cgb.iu.edu/?config=jbrowse%2FvanKessel%2FGSF4066%2Fconfig.json&session=share-PEoXmCIEfG&password=LeDna Zenodo: Perl scripts have been deposited to Zenodo: https://zenodo.org/communities/litr.

## SUPPLEMENTAL MATERIAL

Supplemental figures and tables jb.00042-25-s0001.pdf Fig. S1 to S7 and Tables S1 to S2.

ASM does not own the copyrights to Supplemental Material that may be linked to, or accessed through, an article. The authors have granted ASM a non-exclusive, world-wide license to publish the Supplemental Material files. Please contact the corresponding author directly for reuse.
