# Supplementary material for: Genetic and biochemical analyses reveal direct interactions between LitR and genes important for Vibrio fischeri physiology, including biofilm production
Source: J Bacteriol. 2025 Aug 1;207(8):e00042-25. doi: 10.1128/jb.00042-25 (PMC12369385; doi:10.1128/jb.00042-25)
Supplement: Supplemental figures and tables — Fig. S1 to S7 and Tables S1 to S2. [file jb.00042-25-s0001.pdf]

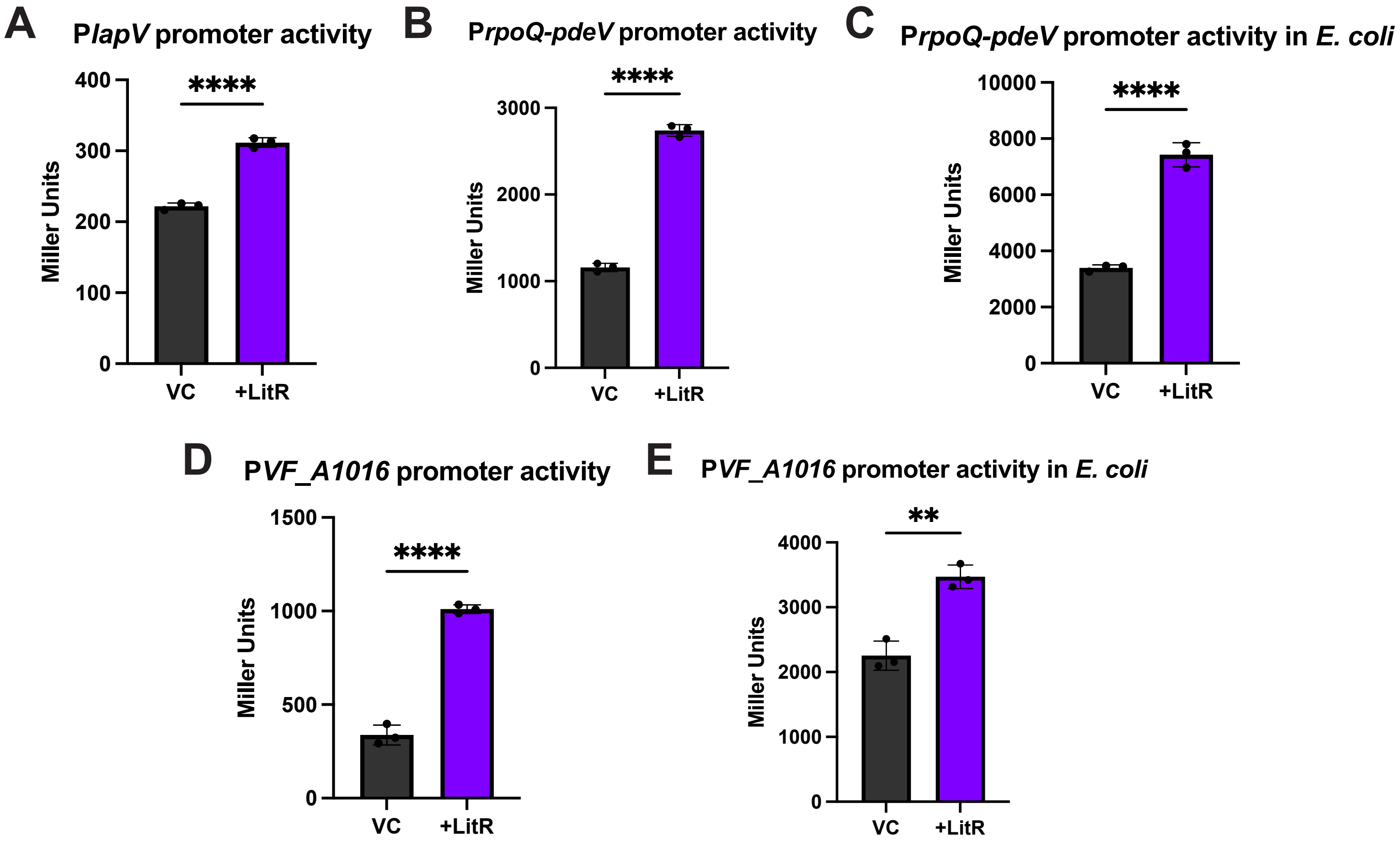

Figure S1. LitR impacts *lapV, rpoQ-pdeV,* and *VF_A1016* promoter activity. Plasmids pVSV105 or pPMF5 were introduced into a strain carrying a Δ*sypQ* mutation and the (A) P*lapV-lacZ* reporter (BF599), (B) P*rpoQ-pdeV-lacZ* reporter (BF298), or (D) P*VF_A1016-lacZ* reporter (BF310) and the resulting strains were grown for 22 h at 24°C with shaking. Cultures were then assessed for B-galactosidase activity as a measure of reporter activity. The plasmids were also introduced to *E. coli* strain MC4100 lambda containing a pJET plasmid expressing (C) P*rpoQ-pdeV-lacZ* or (E) P*VF_A1016-lacZ.* These strains were incubated with shaking at 28°C for 6 h. The resulting cultures were assayed for B-galactosidase activity as a measure of reporter activity. Statistics were performed using an unpaired t-test; **p-value: 0.0019, ****p-value < 0.0001.

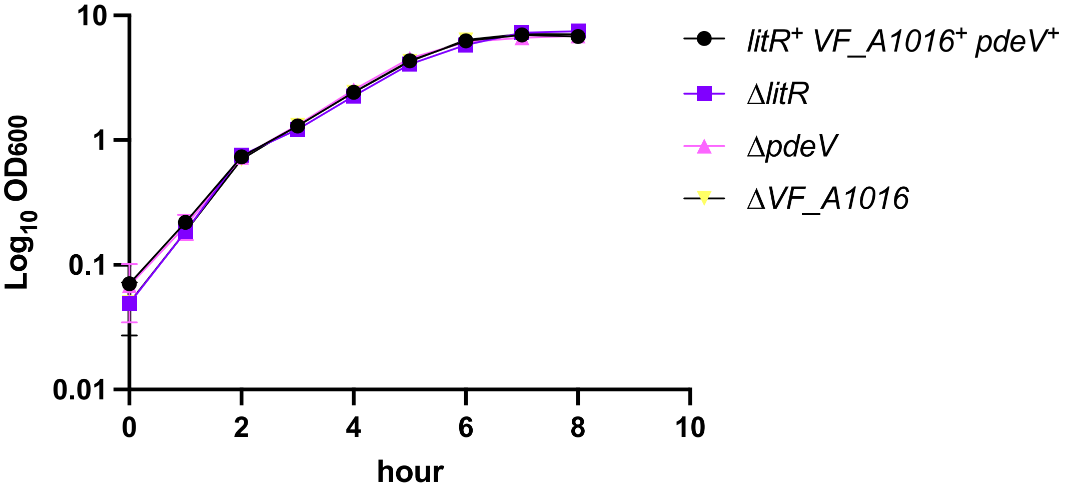

Figure S2. The Δ*pdeV* and Δ*VF_A1016* mutants do not have a growth defect under shaking conditions. The *litR*+ *VF_A1016*+ *pdeV*+ strain (KV9895), the Δ*litR* mutant (BF13), the Δ*pdeV* mutant (KV9410), and the Δ*VF_A1016* mutant (BF84), all carrying a Δ*sypQ* mutation, were grown under shaking conditions for 8 h in LBS + 10 mM $CaCl_2$ at 24°C with OD_{600} measurements taken at each hour. Some error bars cannot be seen because they are shorter than the size of the symbol.

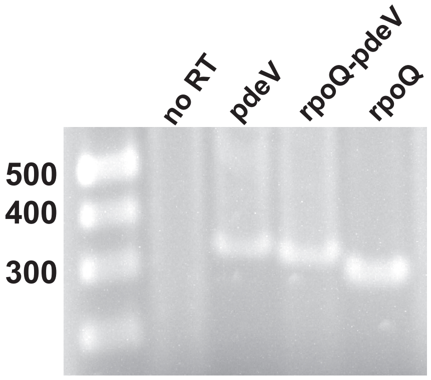

Figure S3. The genes encoding RpoQ and PdeV are in an operon. A DNA agarose gel depicting RT-PCR products amplified from WT (ES114) cDNA as described in Materials and Methods. The "no RT" lane refers to the no reverse transcriptase control, the "pdeV" lane corresponds to cDNA amplified from the coding region of *pdeV*, the "rpoQ-pdeV" lane corresponds to amplified cDNA that includes the intergenic region between *rpoQ* and *pdeV*, and the "rpoQ" lane corresponds to cDNA amplified from the coding region of *rpoQ*.

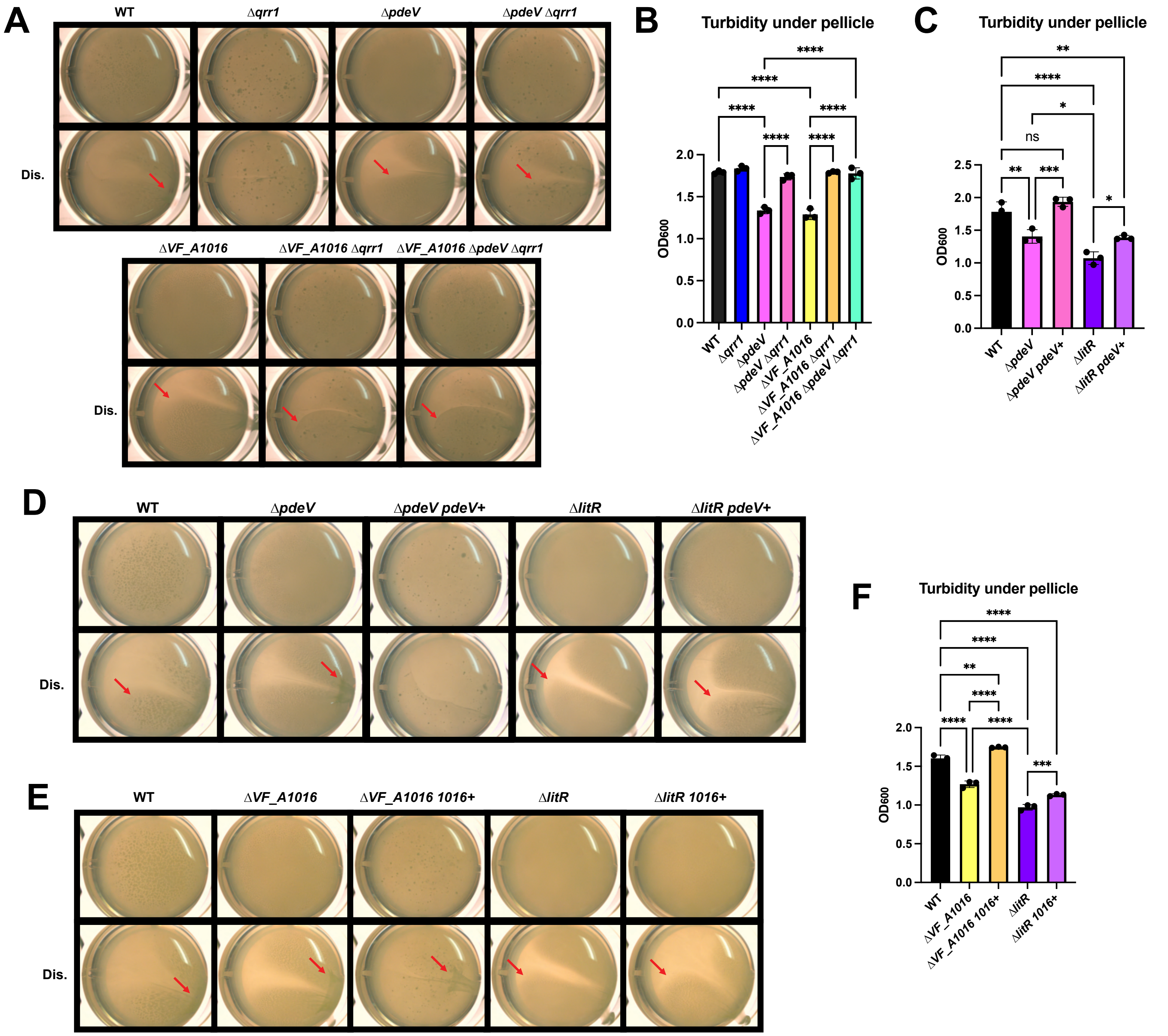

Figure S4. Expression of *VF_A1016* or *pdeV* results in minor suppression of pellicle formation by the Δ*litR* mutant. (A) WT (ES114), the Δ*qrr1* mutant (TIM305), the Δ*pdeV* mutant (KV8969), the Δ*pdeV* Δ*qrr1* mutant (BF647), the Δ*VF_A1016* mutant (BF48), the Δ*VF_A1016* Δ*qrr1* mutant (BF646), and the Δ*VF_A1016* Δ*pdeV* Δ*qrr1* mutant (BF666) or (D) WT (ES114), the Δ*pdeV* mutant (KV8969), the Δ*pdeV pdeV*+ strain (KV9918), the Δ*litR* mutant (KV10494), and the Δ*litR pdeV*+ strain (BF91) or (E) WT (ES114), the Δ*VF_A1016* mutant (BF48), the Δ*VF_A1016 1016*+ strain (BF203), the Δ*litR* mutant (KV10494), and the Δ*litR 1016*+ strain (BF212) were assessed after 72 h of static growth at 24°C in LBS + 10 mM $CaCl_2$. Images were taken with the Zeiss Stemi 2000-c microscope at 6.5x magnification with and without disruption (Dis.) using a toothpick to assess stickiness of the pellicle. Red arrows are used to highlight an area of each disrupted pellicle where stickiness and/or cohesiveness is observed. (B & C & F) The turbidity of the liquid underneath the pellicle was measured by OD_{600} and plotted. Statistics were performed using a one-way ANOVA corrected for multiple comparisons with Tukey's test; ns: not significant, *p-value ≤ 0.0191, **p-value ≤ 0.0061, ***p-value ≤ 0.0009, ****p-value < 0.0001.

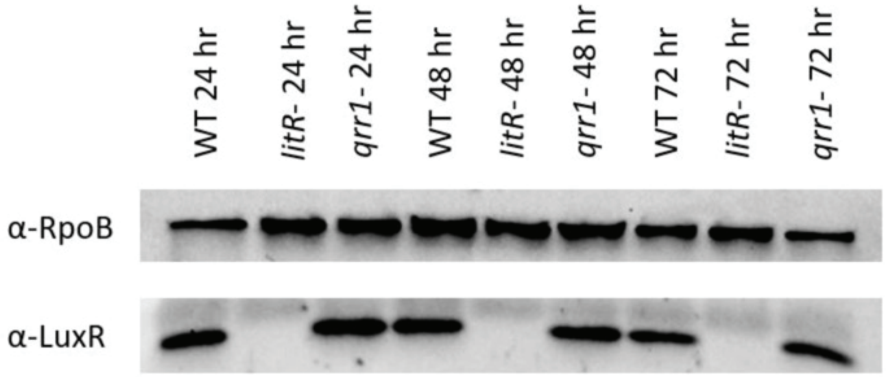

Figure S5. The anti-LuxR^{Vca} antibody can specifically bind to *V. fischeri* LitR. Cell extracts from WT (ES114), the Δ*litR* mutant (KV10494), and the Δ*qrr1* mutant (TIM305) were assessed after 24, 48, and 72 h of shaking growth at 24°C in LBS + 10 mM $CaCl_2$. The samples were assessed for LitR production by Western blot using an antibody against *V. campbellii* LuxR (α-LuxR) with the anti-RpoB antibody (α-RpoB) used as a loading control.

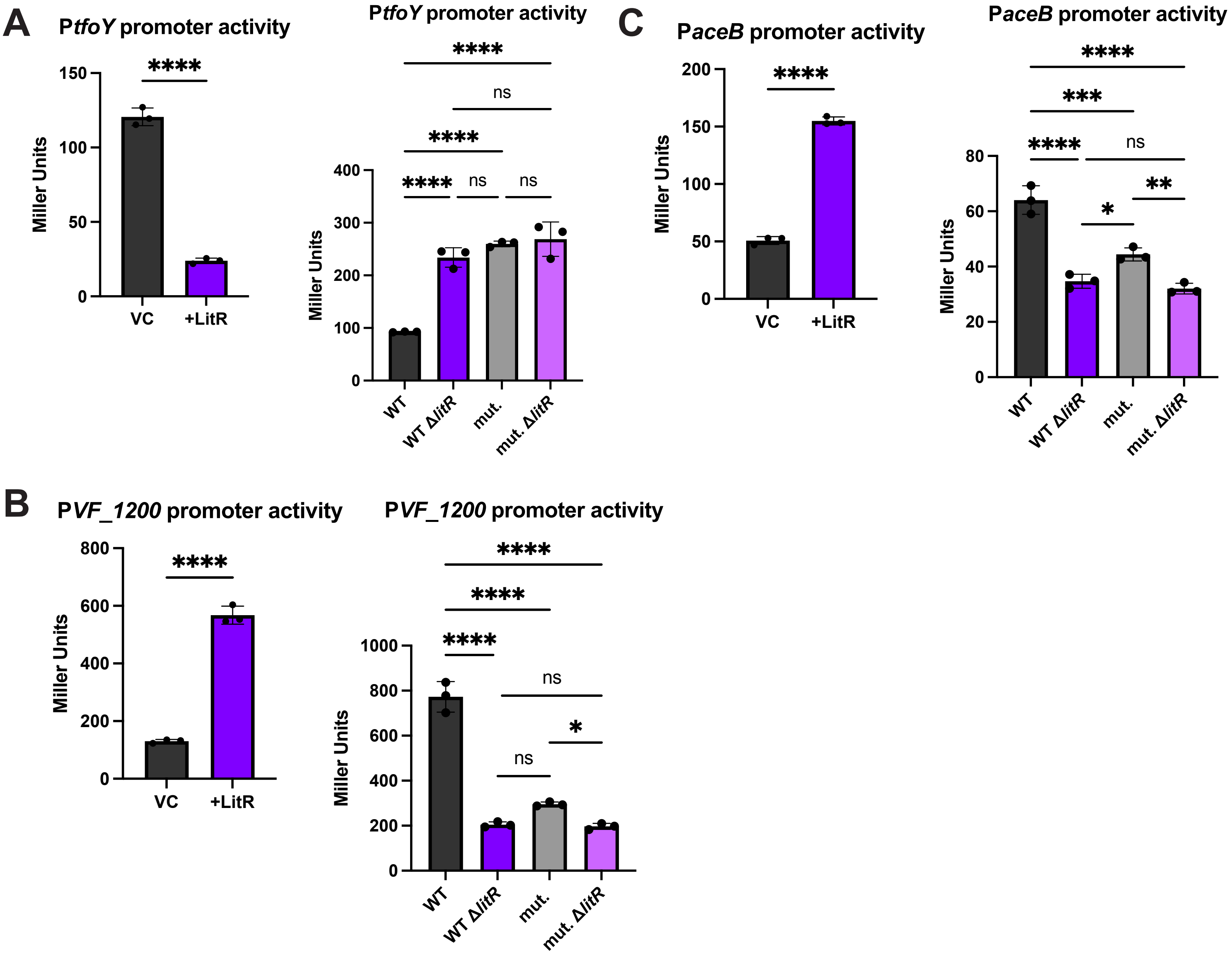

Figure S6. LitR impacts *tfoY*, *VF_1200*, and *aceB* promoter activity. Plasmid pVSV105 or pPMF5 were introduced into strains carrying a Δ*sypQ* mutation and the (A, left) P*tfoY-lacZ* reporter (KV10960), (B, left) P*VF_1200-lacZ* reporter (BF678), or (C, left) P*aceB-lacZ* reporter (KV10957) and the resulting strains were grown for 22 h at 24°C with shaking. Additionally, promoter-*lacZ* fusions in a Δ*sypQ* mutant background were assessed with no change (WT), with a Δ*litR* mutation, with the regulatory region mutated at the predicted LitR binding site based on ChIP data (mut.), and with the regulatory region mutation (mut.) and the Δ*litR* mutation for the (A, right) P*tfoY-lacZ* reporter (KV10945, KV10960, KV11167, KV11168), (B, right) P*VF_1200-lacZ* reporter (KV11163, KV11164, KV11169, KV11170), or (C, right) P*aceB-lacZ* reporter (KV10954, KV10957, KV11165, KV11166) after 22 h at 24°C with shaking. All cultures were assessed for B-galactosidase activity as a measure of reporter activity. Statistics for the left panels were performed using an unpaired t-test; ****p-value < 0.0001. Statistics for the right panels were performed using a one-way ANOVA corrected for multiple comparisons using Tukey's test; ns: not significant, *p-value ≤ 0.0369, **p-value: 0.0071, ***p-value: 0.0004, ****p-value < 0.0001.

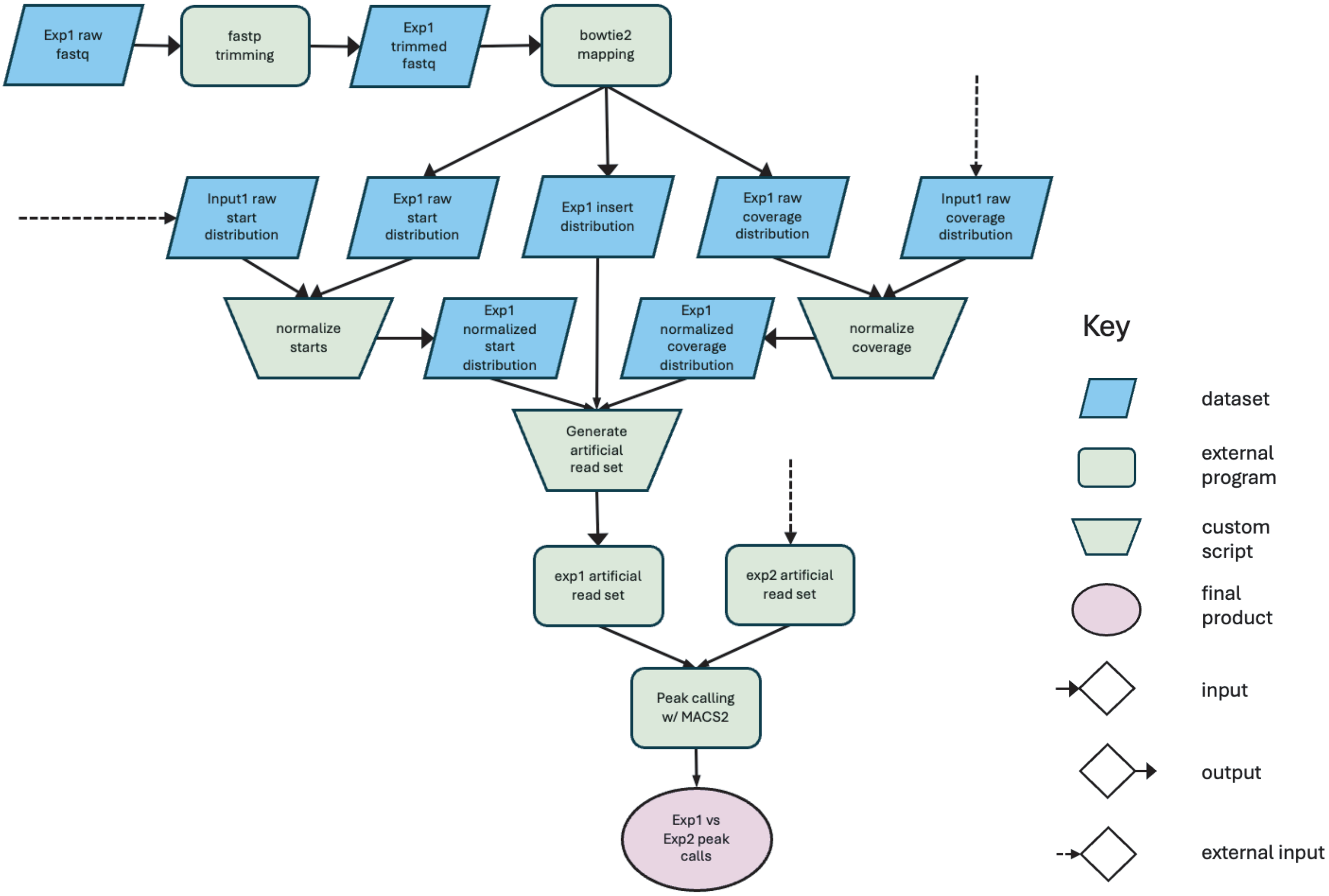

Figure S7. ChIP-seq analysis workflow. Flow chart depicting processing of fastq data to generate normalized artificial distributions and used to perform peak calling. The fastq reads are trimmed and mapped. From the mapping, a distribution of the start sites of the inserts are determined corresponding to the 5' most base pair of the mapped read pair. Additionally, the distribution of the insert coverage is also determined from the mapping. The corresponding input is then used to normalize the experimental insert start distribution using a custom perl script; this normalization removes the over-duplicated DNA associated with the origin of replication in actively growing bacteria. The same normalization is applied to the coverage distribution. Both the normalized start and coverage distribution are used to generate a normalized artificial mapped read insert set for the given experimental sample. These normalized insert sets can be combined using MACS2 to generate peak calls.

Table S1. LitR ChIP-seq peaks

Chromosome	Peak center[1]	Signal Value[2]	Gene(s)[3]	Described/proposed function[4]	Peak location: Coding/Noncoding region[5]
Chromosome I	32101[a]	5.5024	*pqqL*	zinc protease	noncoding
	55733	6.0799	*ubiB*	probable protein kinase	noncoding
	65942	10.6147	*VF_0059*	HEAT repeat domain-containing protein	noncoding
	79730[d]	16.2679	*uvrD <> rarD*	*uvrD* - RecBCD enzyme subunit RecB, *rarD* - Predicted chloramphenicol resistance permease	noncoding
	89723	13.4950	*spf* rRNA	rRNA	noncoding
	99568[b]	6.2861	*VF_0087*	c-di-GMP phosphodiesterase	noncoding
	131733	10.0109	*ompR <> VF_0113*	*ompR*: DNA-binding dual transcriptional regulator, *VF_0113*: Chromosome partitioning protein ParA	noncoding
	179727[a]	5.2001	*gfcE <> VF_0161*	*gfcE*: predicted exopolysaccharide export protein, *VF_0161*: uncharacterized protein	noncoding
	333433	6.6567	*yfbS*	Transporter, divalent anion:sodium symporter family	coding
	369114	6.3649	*uvrA*	UvrABC system protein A, Excinuclease ABC subunit A	coding
	464841	22.3667	*dns*	DNA-specific endonuclease I	noncoding
	539264[b]	5.29148	*yeiM*	nucleoside permease	noncoding
	570032	15.9197	*codA*	cytosine deaminase	noncoding
	656309	19.3020	*VF_0597*	tetratricopeptide repeat protein	coding
	752337	19.8672	*yejE*	Predicted oligopeptide transporter subunit	coding
	759858	8.8954	*glyA*	Serine hydroxymethyltransferase	coding
	774641[a]	7.6102	*luxP*	Autoinducer 2-binding periplasmic protein	coding
	813329[b]	5.7699	*dacA*	serine-type D-Ala-D-Ala carboxypeptidase	noncoding
	863180[b]	7.1516	*yfcH*	Conserved protein with NAD(P)-binding Rossmann-fold domain	coding
	935337[b]	5.6484	*VF_0849*	AMP-(Fatty)acid ligase	coding

	962789[b]	5.3723	*VF_0877*	Transcriptional regulator, LysR family	coding
	983040[b]	7.3794	*VF_0892*	Aspartate 1-decarboxylase	noncoding
	1012198	21.739	*VF_0917* <> *adhE*	*VF_0917*: Cation transporter, VIC family, *adhE*: Aldehyde-alcohol dehydrogenase	noncoding
	1017362[b]	5.7549	*sodB* <> *grxD*	*sodB*: Superoxide dismutase, *grxD*: Glutaredoxin	noncoding
	1055687	9.0626	*tolB*	Tol-Pal system protein TolB	coding
	1107609	7.2460	*VF_1002*	IraD/Gp25-like domain-containing protein	noncoding
	1110844[b]	6.1950	*VF_1005*	ImpA N-terminal domain-containing protein	coding
	1144159	13.8192	*ainS* <> *rluB*	*ainS*: acyl-homoserine-lactone synthase, *rluB*: tRNA pseudouridine synthase A	noncoding
	1161290[b]	6.4835	*VF_1052*	Transporter, divalent anion:sodium symporter family	coding
	1168231	6.0038	*ycgM*	Predicted isomerase/hydrolase	coding
	1174295[b]	10.3917	*VF_1062* <> *VF_1063*	*VF_1062*: Adenylosuccinate synthetase, *VF_1063*: Transcriptional regulator	noncoding
	1175605	28.1482	*gadA*	glutamate decarboxylase	noncoding
	1210045[b]	5.9824	*VF_1095*	nucleoside permease	coding
	1214798[b]	7.3010	*VF_1100*	D-cysteine desulfhydrase-like protein	coding
	1240131	17.7656	*VF_1123*	putative lipoprotein	noncoding
	1253442	6.3086	*VF_1132*	alpha-amylase	noncoding
	1257117	8.4478	*VF_1133* <> *hinT*	*VF_1133*: methyl accepting chemotaxis protein, *hinT*: Purine nucleoside phosphoramidase	noncoding
	1281181	33.2916	*VF_1155* <> *VF_1156*	*VF_1155*: Proton glutamate symport protein, *VF_1156*: Chlorohydrolase/deaminase family protein	noncoding
	1305757[a]	13.4521	*sdaA*	L-serine dehydratase	coding
	1307260	10.6826	*sdaC*	predicted serine transporter	noncoding
	1321111[be]	7.1291	*VF_1190*	uncharacterized iron regulated protein	noncoding

	1334826	17.5182	*VF_1200* <> *VF_2607*	*VF_1200*: diguanylate cyclase, *VF_2607*: uncharacterized protein	noncoding
	1376126[b]	5.4184	*ihfA*	integration host factor subunit alpha	noncoding
	1387564	9.8469	*ompT*	Omptin family protease OmpT	coding
	1392443	12.7440	*VF_1251*	DUF6815 domain-containing protein	coding
	1404789[a]	6.9748	*ssnA*	Predicted chlorohydrolase/aminohydrolase	noncoding
	1424011[b]	8.5789	*prc*	Ribosome maturation factor RimM	coding
	1433648[b]	5.8913	*pyrD*	Dihydroorotate dehydrogenase (quinone)	noncoding
	1443587	7.3862	*VF_1293*	endopeptidase	coding
	1501968	13.1672	*VF_1356*	DUF2971 domain-containing protein	noncoding
	1509195[b]	5.6805	*VF_1363*	VF1363: Formate hydrogenlyase subunit 6	noncoding
	1529441	30.6775	*VF_1380*	penicillin-binding protein	noncoding
	1540785[b]	5.4206	*VF_2614*	copper resistance protein	noncoding
	1565470	5.4787	*mdtK*	Multidrug resistance protein NorM	coding
	1575779[b]	5.3903	*VF_1423*	DUF4381 family protein	coding
	1679140	7.1524	*lapV*	RTX (Repeats in toxin) calcium-binding cytotoxin RtxA1	coding
	1694418	7.1263	*VF_1518*	DnaK-related protein	noncoding
	1705148	7.0448	*VF_1524*	Response regulatory domain-containing protein	coding
	1729709[b]	6.4451	*VF_1546* <> *yjiE*	*VF_1546*: C4 dicarboxylate transporter, *yjiE*: lysR family transcriptional regulator	noncoding
	1737886[b]	5.0675	*nrfA*	Cytochrome c-552, Cytochrome c nitrite reductase	coding
	1762308	7.0832	*tfoY*[c]	DNA transformation protein	noncoding
	1779561	10.5640	*hisP* <> *VF1589*	hisP: Histidinol-phosphate aminotransferase, VF1589: DUF3360 family protein	noncoding
	1801955	9.6628	*cls* <> *VF_1605*	*cls*: Cardiolipin synthase A, *VF_1605*: transporter	noncoding
	1834268	9.9288	*VF_1630* <> *hns*	*VF_1630*: Sodium:proton antiporter, *hns*: Global DNA-binding	noncoding (both),

				transcriptional dual regulator H-NS	coding (hns)
	1889676	6.7285	*yqhD*	alcohol dehydrogenase	coding
	1891805[b]	6.9694	*VF_1679*	DUF4105 domain-containing protein	noncoding
	1908710[f]	12.3457	*truA* > *VF_2628*	*truA*: tRNA pseudouridine synthase A, *VF_2628*: uncharacterized protein	coding
	1933779	27.3125	*VF_1716* <> *VF_1717*	*VF_1716*: AraC family transcriptional regulator, *VF_1717*: transporter, drug/metabolite exporter family	noncoding
	1947046	6.8358	*ygiQ*	putative Fe-S oxidoreductase	noncoding
	1976551	5.4589	*asmA*	predicted assembly protein	coding
	1997407	16.9655	*prkA*	serine kinase	noncoding
	2005953[d]	11.6355	*VF_2633* <> *VF_1777*	*VF_2633*: putative lipoprotein, *VF_1777*: uncharacterized protein	noncoding
	2037316	12.7305	*aroC* <> *VF_2635*	*aroC*: Chorismate synthase, *VF_2635*: uncharacterized	noncoding
	2059097	8.7948	*ccmA*	Cytochrome c biogenesis ATP-binding export protein	noncoding
	2085552	10.3736	*fliE*[c]	Flagellar hook-basal body complex protein	noncoding
	2095888	6.0327	*flaD*[c]	flagellin	noncoding
	2103922	7.0043	*flgK*	flagellar hook-associated protein	noncoding
	2134624	9.3163	*VF_1900* <> *VF_1901*	*VF_1900*: COME operon protein 3, *VF_1901*: DUF2956 domain-containing protein	noncoding
	2141615	43.6125	*narQ*	sensor kinase	coding
	2206684	77.9644	*aceB*	malate synthase	noncoding
	2270790	11.6865	*VF_2042*	methyl accepting chemotaxis protein	noncoding
	2293105	22.7326	*cadB*	Predicted lysine/cadaverine transporter	noncoding
	2299649	16.7546	*dcuB*	C4-dicarboxylase transporter	noncoding
	2314795[b]	7.5768	*flaF*[c]	flagellin	noncoding
	2340310	6.5626	*rpiA*	Ribose-5-phosphate isomerase A	coding
	2346759	6.1842	*yaaJ*	Predicted transporter	noncoding
	2356410	15.4183	*arcA* <> *rrA2*	*arcA*: response regulator, arginine deiminase, *rraA2*:	noncoding

				4-hydroxy-4-methyl-2-oxoglutarate aldolase	
	2404387[b]	7.3034	*VF_2148*	phosphoglucomutase	coding
	2436365[b]	5.3751	*yadB*	Glutamyl-Q tRNA(Asp) synthetase	coding
	2445303	25.3393	*VF_2175*	DUF1127 domain-containing protein	noncoding
	2530646	33.4926	*VF_T0076*	uncharacterized	coding
	2551911	9.2866	*VF_2270*	maltoporin	coding
	2557769[b]	5.96	*cytR*	DNA-binding transcriptional repressor	noncoding
	2562343[b]	5.0691	*yhfA* <> *crp*	*yhfA*: OsmC family protein, *crp*: Transcriptional regulatory protein Fnr	noncoding
	2574623[b]	6.8071	*aroB*	3-dehydroquinate synthase	coding
	2649129	5.1814	*gapA2*	Glyceraldehyde-3-phosphate dehydrogenase	coding
	2667486	5.7811	*VF_2379*	histidine kinase	coding
	2740774	31.8006	*sthA*	Soluble pyridine nucleotide transhydrogenase	coding
	2747938[d]	11.4537	*fliL2*[c] <> *glpG*	*fliL2*: flagellar protein fliL, *glpG*: Predicted intramembrane serine protease	noncoding
	2798330	6.6033	*dsbD*	Thiol:disulfide interchange protein	coding
	2813392	24.288	*VF_2510*	PTS system, mannitol (Cryptic)-specific IIA component	noncoding
	2816720	6.8397	*VF_2513* <> *VF_2512*	*VF_2513*: Cyclic nucleotide binding protein/2 CBS domains, *VF_2512*: SPOR domain-containing protein	noncoding
Chromosome II	12221	9.1441	*VF_A0010*	uncharacterized protein	noncoding
	91325[bf]	5.7243	*dmsD* > *VF_A0084*	dmsD: Chaperone protein TorD, VFA0084: Ferredoxin-type protein NapF	coding
	134908	14.6968	*VF_A0119* <> *VF_A0120*	*VF_A0119*: lactoylglutathione lyase, *VF_A0120*: BmpB	noncoding
	168419	8.8586	*VF_A0152*	diguanylate cyclase	coding
	187665	20.1341	*iutA*	ferric aerobactin receptor	coding
	188754	6.6977	*qmcA*	integral inner membrane protein	noncoding

	194029	19.5477	*VF_A0170*	methyl accepting chemotaxis protein	noncoding
	251829	17.0737	*tadF2*	ATP/GTP-binding site motif A (P-loop) surface protein	coding
	271575[b]	5.2536	*VF_A0244* <> *VF_A0245*	*VF_A0244*: GGDEF/EAL domains protein, *VF_A0245*: uncharacterized protein	noncoding
	413709[a]	6.7871	*VF_A0368*	diguanylate cyclase	noncoding
	423958[b]	5.3443	*VF_A0375*	TonB-dependent receptor	coding
	465915	9.2358	*pyrC*	dihydroorotase	noncoding
	485578[f]	16.3132	*ycaI* > *msbA*	*ycaI*: Recombination protein, *msbA*: ATP-dependent lipid A-core flippase	coding (*ycaI*), noncoding (*msbA*)
	594055	18.1241	*VF_A0517*	WYL domain-containing protein	noncoding
	601729	7.8259	*VF_A0527*	methyl accepting chemotaxis protein	coding
	615002[bf]	5.5117	*VF_A0537* > *VF_A0538*	*VF_A0537*: site-specific DNA-methyltransferase (adenine-specific), *VF_A0538*: Type I restriction-modification system specificity subunit	coding
	631751[b]	5.5877	*intA*	phage family integrase	coding
	635995[b]	5.2232	*VF_A0551*	phosphodiesterase	noncoding
	687986[b]	7.6845	*VF_A0608*	two component response regulator	noncoding
	691848[b]	7.6527	*VF_A0612*	9-hexadecenoic acid cis-trans isomerase	coding
	717788	5.2574	*VF_A0641* <> *VF_A0642*	*VF_A0641*: DUF4156 domain-containing protein, *VF_A0642*: uncharacterized protein	coding
	721499[b]	7.683	*VF_A0646*	N-acetyltransferase domain-containing protein	noncoding
	757099[b]	5.105	*talB*	Transaldolase	noncoding
	762380	5.2856	*VF_A0688* <> *VF_A0689*	*VF_A0688*: HTH cro/C1-type domain-containing protein, *VF_A0689*: uncharacterized protein	noncoding
	794308[a]	5.6674	*VF_A1188* <> *VF_A0709*	*VF_A1188*: uncharacterized protein, *VF_A0709*: protein-N(pi)-phosphohistidine--D-fructose phosphotransferase	noncoding

	814661	5.2551	*VF_A0719*	OmpA-like domain-containing protein	coding
	871007[a]	9.8744	*VF_A0777*	TonB system transport protein ExbD2	coding
	908112	6.512	*VF_A0807*	DUF218 domain-containing protein	coding
	934136	10.7534	*putA*	dual function proline dehydrogenase and proline oxidase	coding
	938059	8.079	*VF_A0833*	transcriptional regulator AraC family	noncoding
	1001875	9.4951	*bcsA*	Cellulose synthase catalytic subunit	coding
	1039544	6.033	*ytfL*	predicted inner membrane protein	noncoding
	1073770[b]	10.3099	*hcpA*	Hydroxylamine reductase	noncoding
	1089228	6.9243	*VF_A0967*	nucleoside permease	coding
	1139454[d]	9.4242	*VF_A1013* <> *pdeV*[c]	*VF_A1013*: Hpt domain-containing protein, *pdeV*: phosphodiesterase	noncoding
	1141786	10.8419	*rpoQ*[c] <> *VF_A1016*	*rpoQ*: RNA polymerase sigma factor, *VF_A1016*: histidine kinase	noncoding
	1168029	11.7799	*sypO*	Chain length regulator (Capsular polysaccharide biosynthesis)	coding
	1214075[a]	6.1489	*VF_A1075*	Possible heptosyltransferase	coding
	1217739[b]	5.3599	*VF_A1078*	Acetyltransferase/hydrolase family protein	coding
	1244651	6.0006	*VF_A1102* <> *VF_A1103*	*VF_A1102*: Site-specific recombinase/phage integrase family, *VF_A1103*: Multidrug resistance protein A	noncoding
	1255173[b]	5.1624	*VF_A1114* <> *VF_A1115*	*VF_A1114*: Cobalt-zinc-cadmium resistance protein CzcD, *VF_A1115*: Transcriptional regulator, LysR family	noncoding
	1258738[a]	5.2246	*VF_A1118*	Conserved protein yaeQ	noncoding
	1265287[b]	6.3772	*VF_A1126*	AraC family transcriptional regulator	coding
	1308674	20.056	*lapI*	Probable lipopolysaccharide assembly protein A	coding
	1317083	10.7019	*lapE*	Outer membrane protein	noncoding

[1]If the peak was present for WT and Δ*qrr1* mutant comparisons to the Δ*litR* mutant, the peak center values were averaged.

[2]A numerical representation of the height and broadness of the peak, with a higher value indicating a taller and narrower peak in relation to the Δ*litR* mutant. If the peak was present for WT and Δ*qrr1* mutant comparisons to the Δ*litR* mutant, the signal values were averaged.
[3]Listed genes are the closest to the peak. Genes that are divergently transcribed are annotated with <> in between. The > annotation refers to genes that are transcribed in the same direction.
[4]Predicted functions were determined based on Uniprot annotations (1).
[5]A determination of whether the peak location (the point in which there are the most reads) is in a coding or noncoding region of the gene.
[a]Peak was only significant in WT.
[b]Peak was only significant in the Δ*qrr1* mutant.
[c]The gene was identified in a *luxO*/phosphomimetic LuxO microarray analysis (2).
[d]Peak was present at the 3' ends of the two genes.
[e]Two peaks were present – one per noncoding region for each of the genes.
[f]The genes are in an operon or have a small intergenic region; the peak is present at the end of one gene and the start of the other.

1. Bateman A, Martin M-J, Orchard S, Magrane M, Adesina A, Ahmad S, Bowler-Barnett EH, Bye-A-Jee H, Carpentier D, Denny P, Fan J, Garmiri P, Gonzales LJDC, Hussein A, Ignatchenko A, Insana G, Ishtiaq R, Joshi V, Jyothi D, Kandasaamy S, Lock A, Luciani A, Luo J, Lussi Y, Marin JSM, Raposo P, Rice DL, Santos R, Speretta E, Stephenson J, Totoo P, Tyagi N, Urakova N, Vasudev P, Warner K, Wijerathne S, Yu CW-H, Zaru R, Bridge AJ, Aimo L, Argoud-Puy G, Auchincloss AH, Axelsen KB, Bansal P, Baratin D, Batista Neto TM, Blatter M-C, Bolleman JT, Boutet E, Breuza L, et al. 2024. UniProt: the universal protein knowledgebase in 2025. Nucleic Acids Research doi:10.1093/nar/gkae1010.
2. Lupp C, Ruby EG. 2005. *Vibrio fischeri* uses two quorum-sensing systems for the regulation of early and late colonization factors. Journal of Bacteriology 187:3620-3629.

Table S2. Additional strains used in this study for strain construction.

Strains	Genotype[1]	Construction	Reference
**BF185**	IG (Erm^r)::P*VF1200-lacZ* Δ*litR*::FRT-$Spec^r$	TT KV9740 with gKV9758	This study
**BF268**	IG (Erm^r)::P*rpoQ-lacZ* Δ*sypQ*::FRT	TT KV9895 with gKV10013	This study
**BF279**	IG::P*VF1200-lacZ*	Erm^r removed from KV9758	This study
**BF281**	IG::P*VF1200-lacZ* Δ*litR*::FRT	$Spec^r$ and Erm^r removed from BF185	This study
**BF285**	IG (Erm^r)::P*rpoQ-lacZ* Δ*sypQ*::FRT Δ*litR*::FRT-$Spec^r$	TT BF268 with gKV9740	This study
**BF291**	IG (Erm^r)::P*VFA1016-lacZ* Δ*sypQ*::FRT	TT KV9895 with gKV10015	This study
**BF529**	IG (Erm^r)::P*nrdR-VFA1016*-P*nrdR-RBS-pdeV-flag*	TT KV10144 with SOE products amplified with primers 3243 & 3025 (ES114) and 2097 & 1487 (KV9482)	This study
**BF594**	IG (Erm^r)::P*lapV-lacZ* Δ*sypQ*::FRT	TT KV9895 with gKV9466	This study
**BF597**	Δ*litR*::FRT-$Spec^r$ IG (Erm^r)::P*lapV-lacZ* Δ*sypQ*::FRT	TT BF594 with gKV9740	This study
**BF651**	Δ*bcsA*::FRT-$Trim^r$ Δ*lapV-1500*::FRT-$Spec^r$	TT KV8616 with gKV10468	This study
**BF665**	Δ*bcsA*::FRT Δ*lapV-1500*::FRT	$Trim^r$ and $Spec^r$ removed from BF651	This study
**JB19**	*litR*::Erm^r	N/A	(1)
**KV7371**	IG::P*sypA-lacZ*	N/A	(2)
**KV8026**	Δ*VFA1016*::FRT-Erm^r	TT ES114 with SOE product amplified with primers 2022 & 2122 (ES114) and 2089 & 2090 (pKV494) and 2123 & 2025 (ES114)	This study
**KV8191**	Δ*sypQ*::FRT-Erm^r	N/A	(3)
**KV8232**	IG::Erm^r-trunc $Trim^r$	N/A	(3)
**KV8408**	*bcsA*::Tn5	N/A	(3)
**KV8613**	Δ*lapV-1500*::FRT-Erm^r	N/A	(4)
**KV8616**	Δ*bcsA*::FRT-$Trim^r$	N/A	(4)
**KV8629**	Δ*lapV-1500*::FRT	Erm^r removed from KV8613	This study
**KV8753**	Δ*sypQ*::FRT-Cm^r Δ*bcsA*::FRT-$Trim^r$	N/A	(4)
**KV8904**	Δ*qrr1* IG::Erm^r-trunc $Trim^r$	TT TIM305 with gKV8232	This study
**KV9220**	Δ*sypA*::FRT-$Spec^r$	TT ES114 with SOE product amplified with primers 1821 & 2808 (ES114) and 2089 & 2090 (pKV521) and 2809 & 423 (ES114)	This study
**KV9324**	Δ*pdeV*::FRT-$Spec^r$	TT ES114 with SOE product amplified with primers 2618 & 2619 (ES114) and 2089 & 2090 (pKV521) and 2620 & 2621 (ES114)	This study
**KV9339**	Δ*rpoQ*::FRT-$Spec^r$	TT ES114 with SOE product amplified with primers 2843 & 2844	This study

		(ES114) and 2089 & 2090 (pKV521) and 2845 & 2846 (ES114)	
**KV9466**	IG (Erm^r)::P*lapV-lacZ*	TT KV7371 with SOE product amplified with primers 2185 & 2090 (pKV502) and 2877 & 2878 (ES114) and 2822 & 2876 (KV7371)	This study
**KV9482**	IG (Erm^r)::P*nrdR-RBS-pdeV-flag*	TT KV8232 with SOE product amplified with primers 2290 & 2892 (KV9563) and 2354 & 1487 (KV9563)	This study
**KV9563**	IG (Erm^r)::P*nrdR-RBS-pdeV*	TT ES114 with gDNA of intermediate strain TT KV8904 with SOE product amplified with primers 2290 & 2090 (pKV506) and 2871 & 2872 (ES114) and 2089 & 1487 (pKV503)	This study
**KV9740**	Δ*litR*::FRT-Spec^r	N/A	(5)
**KV9758**	IG (Erm^r)::P*VF1200-lacZ*	TT KV7371 with SOE product amplified with primers 2185 & 2090 (pKV502) and 2983 & 2984 (ES114) and 2822 & 2876 (KV7371)	This study
**KV10013**	IG (Erm^r)::P*rpoQ-lacZ*	TT KV7371 with SOE product amplified with primers 2185 & 2090 (KV9466) and 3080 & 3277 (ES114) and 2822 & 2876 (KV9466)	This study
**KV10015**	IG (Erm^r)::P*VFA1016-lacZ*	TT KV7371 with SOE product amplified with primers 2185 & 2090 (KV9466) and 3078 & 3276 (ES114) and 2822 & 2876 (ES114)	This study
**KV10111**	IG (Erm^r)::P*nrdR-VFA1016*	TT KV8232 with SOE product amplified with primers 2089 & 2090 (pKV506) and 3265 & 3233 (ES114) and 2196 & 1487 (pKV503)	This study
**KV10144**	IG::P*nrdR-VFA1016*	Erm^r removed from KV10111	This study
**KV10468**	Δ*lapV-1500*::FRT-Spec^r	TT ES114 with SOE product amplified with primers 2224 & 2225 (ES114) and 2089 & 2090 (gKV9220) and 2226 and 2227 (ES114)	This study
**KV10568**	IG (Erm^r)::P*rpoQ*-T1-*lacZ*	TT KV7371 with SOE product amplified with primers 2185 & 2090 (KV10013) and 4328 & 2876 (KV10013)	This study
**KV10593**	Δ*sypQ*::FRT IG (Erm^r)::P*rpoQ*-T1-*lacZ*	TT KV9895 with gKV10568	This study
**KV10619**	Δ*VFA1016*::FRT-Spec^r	TT ES114 with SOE product amplified with primers 1965 & 2122 (ES114) and 2089 & 2090 (pKV521) and 2123 & 1966 (ES114)	This study

**KV10766**	Δ*sypQ*::FRT IG (Erm^r)::P*VFA1016*-T1-*lacZ*	TT KV9895 with gDNA from an intermediate strain made by TT 7371 with SOE product amplified with primers 2185 & 2090 (pKV502) and 4332 & 2876 (KV10015)	This study
**KV10782**	IG (Erm^r)::P*rpoQ*-T2-*lacZ*	TT KV7371 with SOE product amplified with primers 2185 & 2090 (pKV502) and 4438 & 2876 (KV10013)	This study
**KV10793**	IG (Erm^r)::P*VFA1016*-T2-*lacZ*	TT KV7371 with SOE product amplified with primers 2185 & 2090 (pKV502) and 4470 & 2876 (KV10015)	This study
**KV10797**	Δ*sypQ*::FRT IG (Erm^r)::P*rpoQ*-T2-*lacZ*	TT KV9895 with gKV10782	This study
**KV10798**	Δ*sypQ*::FRT IG (Erm^r)::P*VFA1016*-T2-*lacZ*	TT KV9895 with gKV10793	This study
**KV10848**	IG (Erm^r)::P*VFA1016*-T3-*lacZ*	TT KV7371 with SOE product amplified with primers 2185 & 2090 (pKV502) and 4491 & 2876 (KV10015)	This study
**KV10850**	IG (Erm^r)::P*rpoQ*-T3-*lacZ*	TT KV7371 with SOE product amplified with primers 2185 & 2090 (pKV502) and 4330 & 2876 (KV10013)	This study
**KV10859**	Δ*sypQ*::FRT IG (Erm^r)::P*VFA1016*-T3-*lacZ*	TT KV9895 with gKV10848	This study
**KV10861**	Δ*sypQ*::FRT IG (Erm^r)::P*rpoQ*-T3-*lacZ*	TT KV9895 with gKV10850	This study
**KV10910**	IG (Erm^r)::P*rpoQ*-*rpoQ*	TT KV8232 with SOE product amplified with primers 2290 & 2090 (pKV502) and 3080 & 2619 (ES114) and 2196 & 1487 (pKV503)	This study
**KV10944**	IG (Erm^r)::P*aceB*-*lacZ*	TT KV7371 with SOE product amplified with primers 2185 & 2090 (pKV502) and 4510 & 4511 (ES114) and 2822 & 2876 (KV9466)	This study
**KV10951**	Δ*sypQ*::FRT Δ*litR*::FRT-$Spec^r$	TT KV9895 with gKV9740	This study
**KV11120**	IG (Erm^r)::P*aceB*-mutated-*lacZ*	TT KV7371 with SOE product amplified with primers 2185 & 4545 (KV10957) and 4544 & 2876 (KV10957)	This study
**KV11136**	IG (Erm^r)::P*tfoY*-mutated-*lacZ*	TT KV7371 with SOE product amplified with primers 2185 & 4541 (KV10960) and 4540 & 2876 (KV10960)	This study
**KV11137**	IG (Erm^r)::P*VF1200*-mutated-*lacZ*	TT KV7371 with SOE product amplified with primers 2185 & 4547 (BF185) and 4546 & 2876 (BF185)	

[1]Abbreviations: IG, gene inserted at intergenic region between genes *yeiR* and *glmS* along with an FRT scar, with one exception: KV7371 does not contain an FRT scar; IG (Erm), gene inserted between *yeiR* and *glmS* along with FRT-Erm^r; TT, TfoX-mediated transformation using *tfoX*-overexpressing version of indicated strain; trunc, truncation; RBS, idealized ribosome binding site; FLAG, FLAG-epitope tagged; FRT, Flippase Recognition Target; if not followed by an antibiotic resistance gene, then the antibiotic cassette was flipped out leaving an FRT scar within the chromosome.

1. Bose JL, Kim U, Bartkowski W, Gunsalus RP, Overley AM, Lyell NL, Visick KL, Stabb EV. 2007. Bioluminescence in *Vibrio fischeri* is controlled by the redox-responsive regulator ArcA. Molecular Microbiology 65:538-553.
2. Norsworthy AN, Visick KL. 2015. Signaling between two interacting sensor kinases promotes biofilms and colonization by a bacterial symbiont. Molecular Microbiology 96:233-248.
3. Visick KL, Hodge-Hanson KM, Tischler AH, Bennett AK, Mastrodomenico V. 2018. Tools for rapid genetic engineering of *Vibrio fischeri*. Applied and Environmental Microbiology 84:AEM.00850-18.
4. Christensen DG, Marsden AE, Hodge-Hanson K, Essock-Burns T, Visick KL. 2020. LapG mediates biofilm dispersal in *Vibrio fischeri* by controlling maintenance of the VCBS-containing adhesin LapV. Molecular Microbiology 114:742-761.
5. Fung BL, Visick KL. 2025. LitR and its quorum-sensing regulators modulate biofilm formation by *Vibrio fischeri*. Journal of Bacteriology doi:10.1128/jb.00476-24.
